# Indirect (implicit) and direct (explicit) self-esteem measures are virtually unrelated: A meta-analysis of the initial preference task

**DOI:** 10.1371/journal.pone.0202873

**Published:** 2018-09-06

**Authors:** Jakob Pietschnig, Georg Gittler, Stefan Stieger, Michael Forster, Natalia Gadek, Andreas Gartus, Krisztina Kocsis-Bogar, Bettina Kubicek, Marko Lüftenegger, Jerome Olsen, Roman Prem, Nina Ruiz, Benjamin G. Serfas, Martin Voracek

**Affiliations:** 1 Department of Applied Psychology: Health, Development, Enhancement and Intervention, Faculty of Psychology, University of Vienna, Vienna, Austria; 2 Department of Psychology, University of Konstanz, Konstanz, Germany; 3 Department of Psychology, Karl Landsteiner University of Health Sciences, Krems, Austria; 4 Department of Basic Psychological Research and Research Methods, Faculty of Psychology, University of Vienna, Vienna, Austria; 5 Department of Applied Psychology: Work, Education and Economy, Faculty of Psychology, University of Vienna, Vienna, Austria; Sapienza, University of Rome, ITALY

## Abstract

**Background:**

The initial preference task (IPT) is an implicit measure that has featured prominently in the literature and enjoys high popularity because it offers to provide an unobtrusive and objective assessment of self-esteem that is easy to administer. However, its use for self-esteem assessment may be limited because of weak associations with direct personality measures. Moreover, moderator effects of sample- and study-related variables need investigation to determine the value of IPT-based assessments of self-esteem.

**Methods:**

Conventional and grey-literature database searches, as well as screening of reference lists of obtained articles, yielded a total of 105 independent healthy adult samples (*N* = 17,777) originating from 60 studies. Summary effect estimates and subgroup analyses for potential effect moderators (e.g., administration order, algorithm, rating type) were calculated by means of meta-analytic random- and mixed-effects models. Moreover, we accounted for potential influences of publication year, publication status (published vs. not), and participant sex in a weighted stepwise hierarchical multiple meta-regression. We tested for dissemination bias through six methods.

**Results:**

There was no noteworthy correlation between IPT-based implicit and explicit self-esteem (*r* = .102), indicating conceptual independence of these two constructs. Effects were stronger when the *B*-algorithm was used for calculation of IPT-scores and the IPT was administered only once, whilst all other moderators did not show significant influences. Regression analyses revealed a somewhat stronger (albeit non-significant) effect for men. Moreover, there was no evidence for dissemination bias or a decline effect, although effects from published studies were numerically somewhat stronger than unpublished effects.

**Discussion:**

We show that there is no noteworthy association between IPT-based implicit and explicit self-esteem, which is broadly consistent with dual-process models of implicit and explicit evaluations on the one hand, but also casts doubt on the suitability of the IPT for the assessment of implicit self-esteem on the other hand.

## Introduction

Personality psychology has been striving in the past to develop measures that are unobtrusive (i.e., objective), easy to administer, and yet provide valid assessments of traits. Such ideas can be traced back to at least the 1960s (e.g., [1]) and continue to receive increasing attention in the literature.

One approach that has been proposed to satisfy this demand emerged in the form of implicit personality tests. Particularly, the development of indirect measures for self-esteem received considerable attention, resulting in the adoption of a substantial number of alleged proxies for the measurement of implicit self-esteem. This includes the preference for initials and name letters as opposed to non-name letters [2], general name liking [3], signature height (e.g., [4]), or procedurally more demanding reaction time-based measures, such as the implicit association test [5].

To date, a consensus has yet to be reached in terms of the conceptual nature of both implicit and explicit self-esteem. Specifically, two broad types of theoretical models have been proposed to explain the implicit and explicit self-esteem associations: Common-core and dual-process models (for an overview, see [6]). On the one hand, in the common-core model, it is assumed that scores on both implicit and explicit self-esteem measures are expressions of one common latent dimension which is assessed in different ways [7,8]. This common dimension can be either assumed (i) to be measured equally well by direct and indirect measures, although different aspects are captured (i.e., the equal relationship hypothesis) or (ii) to represent more accurate (i.e., implicit self-esteem) or less accurate (i.e., explicit self-esteem) assessments of this common-core (i.e., the hierarchy hypothesis; for a review, see [8]).

On the other hand, in dual-process models implicit and explicit self-esteem are viewed as expressions of different largely independent cognitive processes (e.g., [9,10]). Whilst implicit evaluations are mainly linked to impulsive and associative processes in these models, explicit evaluations are assumed to relate to reflective processes which are informed by knowledge and beliefs [10].

These different models lead to different expectations for explicit and implicit self-esteem associations. Whilst in common-core models a substantial association between these two constructs is expected, dual-process models suggest only weak correlations.

An approach that has gained particular popularity for the assessment of implicit self-esteem is the initial preference task (IPT), as popularized by the procedure of Kitayama and Karasawa [2], perhaps owing to the comparative ease of administration (i.e., no necessity to measure reaction time and therefore computer-based assessment). The IPT has been developed based on the pioneering work of Nuttin [11,12] who noticed that individuals tend to prefer letters of their name and particularly their initial name letters over other letters of the alphabet. Systematic differences in the liking of the name letters have been attributed to differing amounts of self-worth (i.e., implicit self-esteem), thus representing an indirect measure for the assessment of self-esteem. Subsequently, this name letter effect has been popularized in the scientific literature as a means to assess individuals’ self-esteem that is less susceptible to socially desirable responding or impression management than traditional self-report measures are.

IPT-based assessments of implicit self-esteem continue to enjoy considerable popularity in the literature. However, despite this, several conceptual questions about the IPT remain unanswered, particularly in terms of its usefulness for the assessment of self-esteem. In the extant literature, IPT scores are frequently treated as an indicator for the “self-evaluative climate” (i.e., representing a trait), whilst explicit self-esteem scores are considered to be an indicator for the “self-evaluative weather” (i.e., representing a state; [13], p.157). Following this interpretation as well as its original conceptualization, implicit and explicit self-esteem should be related at least to some degree.

However, an initial, now increasingly outdated, meta-analysis based on a comparatively small number of samples (*k* = 19) showed that correlations between IPT-based scores and explicit self-esteem were small (*r* = .12, 95% CI: .089 to .142; [14], p.529; although this has been interpreted as evidence for modest associations) and evidence from novel primary studies indicates that IPT-based assessments may be poor measures of self-esteem altogether [15]. One interesting point that emerged from this first meta-analytic account is that correlations between indirect (implicit) and direct (explicit) measures were somewhat stronger when explicit self-esteem measures were administered before implicit ones. This pattern suggests that the accessibility of self-esteem (as facilitated by exposure to explicit measures) may impact responses on the IPT [16]. Similarly, evidence from other meta-analytic and multi-study investigations indicates that associations between the IPT and explicit self-esteem measures are small at best [17,18].

One point that so far has been insufficiently addressed is whether dissemination bias (i.e., publication or reporting bias, *p*-hacking, effect strength in published vs. unpublished reports) or the decline effect [19] may have led to inflated summary effect sizes. Another point of interest is whether the use of different algorithms, which have been proposed to obtain IPT-scores (i.e., controlling for different kinds of response tendencies; for an overview, see [20]), moderates the association between implicit and explicit measures.

### The present meta-analysis

There are a number of reasons, which make it clearly necessary to update the meta-analysis of Krizan and Suls [14]. First, within the decade that has passed since its publication (in fact, only studies published until 2005 were included in [14]), publication output pertaining to IPT-based assessments has substantially increased. We included more than five times more samples in the present meta-analysis (refer to our Final Sample section) compared to this past meta-analysis. Second, the development of novel meta-analytic methods in terms of both summary effect estimation as well as bias diagnostics (e.g., *p*-curve, *p*-uniform [21–24]) allows for a more precise assessment of the stability of effects, particularly in cases of weak or volatile associations, such as here.

Third, theoretical considerations [19] as well as recent empirical accounts [25,26] convergently suggest that systematic time trends in empirical effects should lead to a (continuous) decline of effect sizes over time. Should such a mechanism affect the presently investigated association between the IPT and direct measures of self-esteem, it is likely that the previously observed borderline non-trivial association [14] may decrease even more. Thus, our meta-analysis may contribute in determining the explanatory value of common-core and dual-process theories.

However, even if a borderline weak effect holds up in the meta-analysis, this finding would be remarkable because in psychology “everything correlates to some extent with everything else” (e.g., [27]). Even when keeping the expectations of dual-process models in mind [10], it would seem noteworthy if the conceptually close constructs of IPT-based implicit and explicit self-esteem were only to correlate to such a minor degree, because even in those models some allowance is being made for relations of implicit and explicit self-esteem (e.g., [28]).

Consequently, here we investigate evidence for associations between IPT-based implicit self-esteem and explicit self-esteem measures across a large number of healthy adult samples. Moreover, we re-examine moderator effects of administration order (i.e., initial assessment of explicit vs. implicit self-esteem) and provide novel evidence about IPT exposure effects (whether the IPT had been administered once or twice to each participant: single vs. double administration of the IPT), the algorithm used for the calculation of IPT scores, rating type (letter liking vs. attractiveness), participant sex, and publication status (published vs. unpublished data source). In ancillary analyses, we provide summary effects for the correlations with first and last initials separately. Finally, we provide evidence for potential dissemination bias and the decline effect in the IPT literature.

## Methods

### Implicit self-esteem measure

The IPT is a self-report measure which requires participants to rate their liking of the letters of the alphabet (sometimes interspersed with numbers or ASCII symbols) on a Likert-typed scale (ranges: min. 5 to max. 9 responses in the present meta-analysis, except for 2 samples where letters where rated on a scale from -10 to +10). Conceptually, this should provide an implicit (i.e., unobtrusive) measure of self-esteem according to the congruence of initial name letter liking or attractiveness with the preferred name letters. More positive evaluations of (initial) name letters as compared to non-name letters reflect higher implicit self-esteem. One advantage of the IPT is that it can be administered as either pen-and-paper or computer test, whilst reaction time-based implicit tests, such as the Implicit Association Test (IAT; [5]), typically (but not exclusively) mandate computer administration and require specialized software.

Typically, IPT scores are calculated according to one of several published algorithms (below, we describe the procedure for initial letters only, although all of these algorithms may be used to examine all name letters as well; for a detailed overview, refer to [17]). Specifically, preferences for initial name letters, as compared to non-name letter can be calculated according to the (i) *B*-algorithm: differences between individual liking of name initials and average liking of participants who do not have these initial name letters, (ii) *S*-algorithm: differences between individual liking of name initials and average individual non-initial letter liking, (iii) *D*-algorithm: differences between individual liking of name initials and average liking of participants who do not have these initial name letters, subsequently divided by average individual non-initial letter liking (i.e., representing a combination of the first two algorithms, thus controlling for both between- and within-individual response tendencies), (iv) *I*-algorithm: differences between ipsatized individual initial ratings (i.e., following the approach of the *B*-algorithm) and ipsatized baseline letter liking, therefore controlling for both baseline letter likeability and individual response tendencies, (v) *Z*-algorithm: differences between *z*-transformed individual initial liking (i.e., following the approach of the *B*-algorithm) and *z*-transformed average liking of participants who do not have these initial name letters, and (vi) *R*-algorithm: differences between individual corrected (i.e., based on a regression-based approach, accounting for general letter liking and response tendencies) name letter liking and average non-name letter liking (see, [29] for a detailed description of this approach).

Although these different algorithms are based on similar ideas, they account for differing sources of systematic error variance. In fact, psychometric evaluations of and comparisons between five of these algorithms suggest that the *I*-algorithm typically shows the best psychometric properties. In 18 independent investigations that scrutinized the adequacy of five different scoring algorithms (i.e., *B*-, *S*-, *D*-, *I*-, and *Z*-algorithms), internal consistencies and split-half reliabilities were highest for the *I*- and *S*-algorithm (average Cronbach α = .47 and .48, respectively), whilst the score distributions for the *I*-algorithm yielded the smallest number of skewed distributions [17]. Consequently, it has been recommended as the method of choice when scoring the IPT (e.g., [18]), although the use of the *I*-algorithm has not remained uncriticized (e.g., [20]). However, as will be seen from our data, the *B*-algorithm seems to be the most often adopted approach for the calculation of IPT scores (refer to our sample description).

### Literature search

First, a cited reference search for Nuttin J* (1985) AND (1987) was performed in the ISI Web of Knowledge database. This strategy was considered useful because primary studies investigating associations between explicit self-esteem with IPT scores may be expected to cite the originally published paper that introduced the name letter effect. Second, we searched the Open Access Theses and Dissertation database (www.oatd.org [30]) for the keywords “implicit self-esteem” to identify potentially includable studies from the grey literature. All relevant results from databases up to December 2016 were included. Finally, we screened the reference lists of obtained full-text articles to assess further studies that might have been missed.

**Fig 1:**
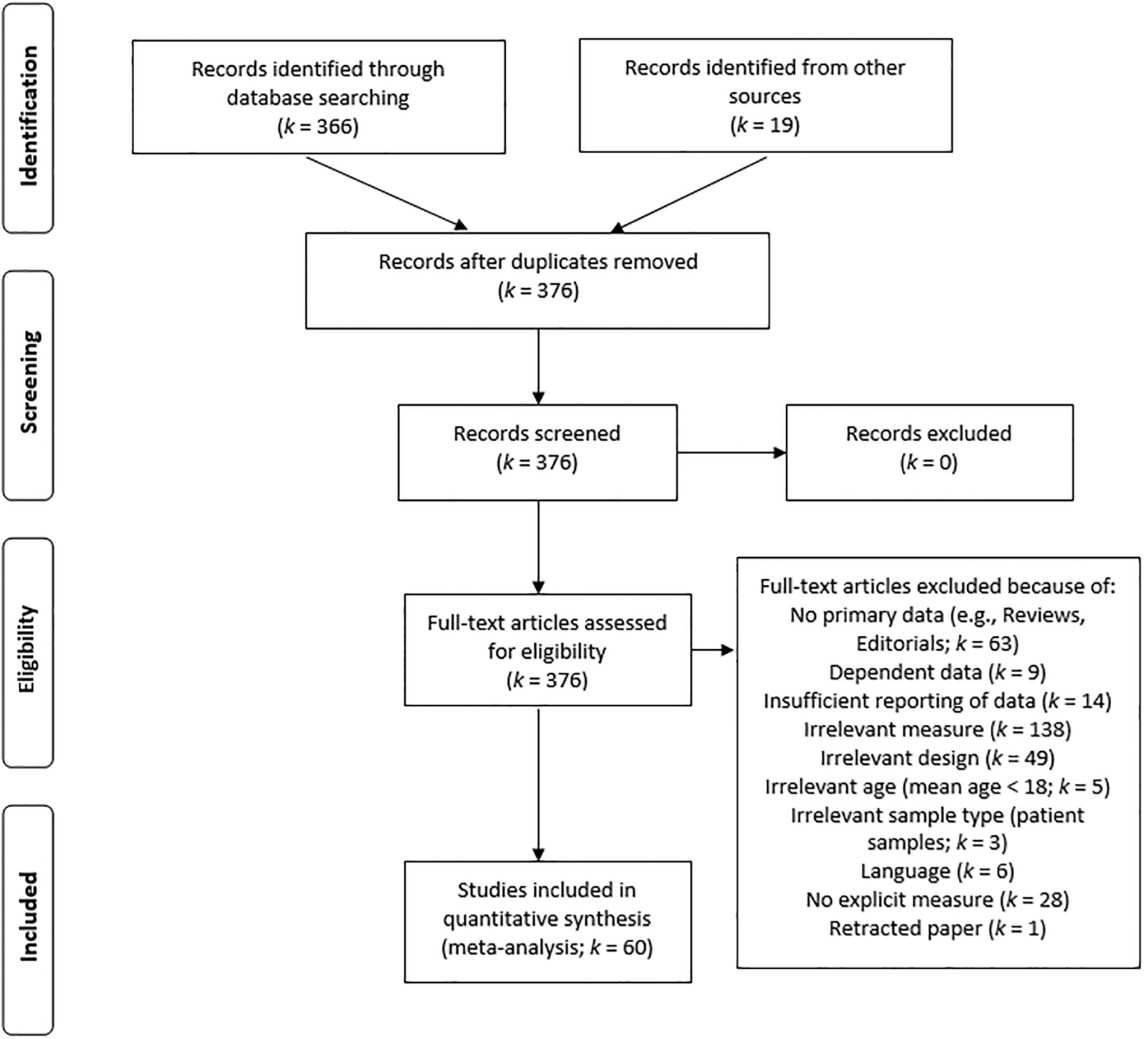
PRISMA flowchart for study retrieval, eligibility, and inclusion of primary studies in the meta-analysis.

When studies met all other inclusion criteria (see below), but did not report sufficient statistical parameters to derive the effect size of interest, the missing parameters were requested by email from the corresponding authors of primary studies. If no response was received within two weeks, a reminder was sent. All data that were received until three weeks after the initial email to the study authors were included in the present analyses (totaling 5 studies, *k* = 14; see documentation in S1 Text and S1 Table). A flowchart of the literature search process according to the PRISMA guidelines is shown in Fig 1 and a list of included and excluded references is provided in the Online Supplement (S1 Text).

### Inclusion criteria

Primary studies had to meet four criteria to be included in the present meta-analysis. First, zero-order correlations between the IPT and a measure of explicit self-esteem had to be reported. Second, samples had to be from adult (i.e., mean age > 18 years) and healthy populations. Third, articles had to be published in English, French, German, or Spanish. Finally, the reported data had to be independent from other included studies. In cases of data dependencies or sample overlap (i.e., if it was determined that data from identical or largely identical samples had been published in different publications), only one effect was retained: the preference for inclusion was based on effect sizes of published data, larger samples, and more recent study years.

### Coding

Potentially includable studies were initially coded independently by two researchers respectively [JP and one of authors four to 13 as well as six further coders; 41% of screened studies] or twice by the same researcher [JP]. Correlation coefficients, sample sizes, sample percentage of men, mean participant ages, and publication years were recorded, and primary studies were coded into categories according to IPT exposure (single vs. double administration of the IPT), administration order of measures (implicit first, explicit first, randomized, counterbalanced, unclear; this was supplemented by information obtained via personal communications by the third author [SS] in the course of another meta-analysis; [18]), publication status (published vs. unpublished studies), rating type (liking vs. attractiveness), and the algorithm used to compute initial preference scores. Whenever correlations from multiple algorithms were reported, we included the results for the most frequently reported algorithms (which are, in descending order: the *B*-, *I*-, *R*-, *Z*-, *S*-, and *D*-algorithm; see above for a description of these different algorithms). In few cases, correlations of first and last name initials with explicit self-esteem were provided only separately (*k* = 12). In these cases, we averaged correlation coefficients to obtain an estimate for overall IPT correlations with explicit measures for our main analysis (we provide summary effect estimates based on first and last name correlations in a supplementary analysis based on *k* = 18 samples). Coding discrepancies were resolved through discussion with an independent third coder [GG].

### Data analysis

Prior to all analyses, correlation coefficients were transformed to Fisher *Z*s and were subsequently backtransformed for ease of interpretation, following standard practice for *r*-based meta-analyses. Initially, we used random-effects estimators for the calculations of (subgroup) summary effect sizes. We consciously decided against using fixed-effect models because of the expected functional non-equivalence of studies (i.e., included primary studies were from many independent researchers that applied different designs; e.g., [31], pp. 83–84). Then, subgroup analyses were performed using a mixed-effects approach. In further analyses, weighted stepwise hierarchical multiple weighted meta-regressions were calculated to assess influences of moderator variables, including estimates for possible effect decreases due to the decline effect [19]. Finally, we used sensitivity analyses to account for potential summary estimate-biasing effects of large individual samples (i.e., by estimating summary effects, whilst omitting one individual effect size within *k* turns).

#### Dissemination bias

It should be acknowledged that many of the identified studies did not focus on the assessment of the relationship between the IPT and explicit self-esteem as their primary goal, which makes the occurrence of “traditional” publication bias less likely (i.e., non-publication of entire studies). However, summary effect-inflating mechanisms of selective reporting (i.e., systematic omissions of non-significant results in published studies) are well-documented [32,33] and were expected to be the main source of bias (if any) in the present meta-analysis.

We used seven methods to detect different forms of dissemination biases. For all these calculations, data were restricted to published results only (i.e., no data from unpublished studies or personal communications were included). First, we visually inspected funnel plot asymmetry [33]. Second, Begg and Mazumdar’s [34] rank-order correlation method was used. Within this approach, effect-size estimates are correlated with sampling variances which should not yield significant associations in the absence of publication bias. Third, Sterne and Egger’s [35] regression approach was used to investigate influences of study precision on the standard normal deviate of the effect size (i.e., effect sizes divided by their standard errors). Within this approach, the regression intercept should not differ significantly from zero in cases of no publication bias. Fourth, trim-and-fill analyses were calculated, which provide adjusted estimates for fixed-effect- or random-effects-based calculations, as well as the numbers of imputed missing studies, based on funnel plot asymmetry [36]. Fifth, we calculated excess significance estimates following the approach of Ioannidis and Trikalinos [37]. In this test, the expected number of significant results (based on the power of individual studies when referenced to the summary effect) is compared to the number of observed significant effects with hypothesis-conforming signs.

Finally, we used two recently developed detection methods for dissemination bias (namely, *p*-curve and *p*-uniform) that are based on the observed distributions of published significant *p*-values (i.e., *p*s < .05). Because only published and nominally significant values are considered, this makes effect estimates arguably insensitive to non-retrievable unpublished (and therefore likely non-significant) results and makes it possible to assess more insidious forms of dissemination bias such as *p*-hacking (e.g., repeated calculation of significance statistics in primary studies by means of different methods or inclusion of different a posteriori-selected covariates, until nominal significance is achieved).

The idea of *p*-curve [21] is to compare the observed distributions of significant *p*-values to the expected distribution of *p*-values in the presence of a null effect (i.e., a uniform distribution of *p*-values). In the presence of a non-zero effect, *p*-value distributions should be significantly right-skewed, which can be assessed by either binomial tests (i.e., by comparing the number of *p*-values < .025 with those ranging from .025 to .050) or continuous tests. The evidential value of a study set can be assessed by evaluating whether the observed *p*-value distribution is flatter than the theoretical *p*-value distribution at 33% power. Finally, effect sizes can be estimated by minimizing a loss function, thus yielding a curve (with a certain effect size associated to it), that most closely resembles the observed *p*-curve (for detailed description, see [22]). This is possible because for a certain statistical test (or effect-size metric), the *p*-curve is a function of the sample size and the underlying true population effect.

A similar idea has led to the development of the *p*-uniform method [23], which allows assessment of *p*-hacking by comparing the distribution of conditional *p*-values (i.e., based on the population effect size) with a uniform distribution. The population effect is estimated by obtaining the summary effect size that fits closest to a conditional *p*-value distribution. In a similar manner, confidence intervals for the summary effect are calculated. Moreover, a significance test of the population effect can be obtained by comparing the observed *p*-value distribution with a uniform distribution.

Two limitations of these two methods should be noted though. On the one hand, both *p*-curve and *p*-uniform have been developed in the context of fixed-effect models and have been shown to systematically overestimate summary effects in presence of moderate to large between-study heterogeneity [24]. On the other hand, these methods are unsuitable for inclusion of *p*-values that are associated with effect sizes showing differing signs. Therefore, prior to data analyses, we decided to use only *p*-values associated with positive effect sizes (i.e., hypothesis-conforming values), to provide an upper threshold of the population effect. All analyses were performed in SPSS, the open Source Software R [38] by means of the package metafor [39], the online application *p*-curve (www.p-curve.com [40]), and the *p*-uniform web application (available from https://rvanaert.shinyapps.io/p-uniform/). We provide the code for all our calculations in the Online Supplement (S2 Text), excepting effect size estimation by means of *p*-curve (the R-code is available from www.p-curve.com [40]).

### Final sample

In all, data from 60 studies comprising 105 independent healthy adult samples (*N* = 17,777; 35.4% men) were included in the meta-analysis. Mean ages of participants ranged from 18.4 to 37.7 years (weighted mean age = 25.1), and participants were from 14 different countries (Australia, Austria, Belgium, Bulgaria, Canada, Colombia, Germany, the Netherlands, Romania, Serbia, Singapore, Spain, UK, USA). The IPT was administered in pen-and-paper format (*k* = 42) or on a computer (*k* = 58; in two samples, both administration types were used and for three samples the administration type was unclear). In all, 62 samples used the *B*-, 26 the *I*-, 3 the *R*-, 3 the *Z*-, and 2 the *S*-algorithm to calculate IPT scores (nine samples either used different approaches or the exact utilized algorithm was unclear). For the assessment of explicit self-esteem, all studies used self-report questionnaires, with the Rosenberg Self-Esteem Scale (*k* = 90) being the most frequently reported measure. Characteristics and correlation coefficients of all included samples are detailed in the Online Supplement (S1 Table). A checklist detailing our meta-analytic outline according to the PRISMA guidelines [41] is available in the Online Supplement (S2 Table).

## Results

**Fig 2:**
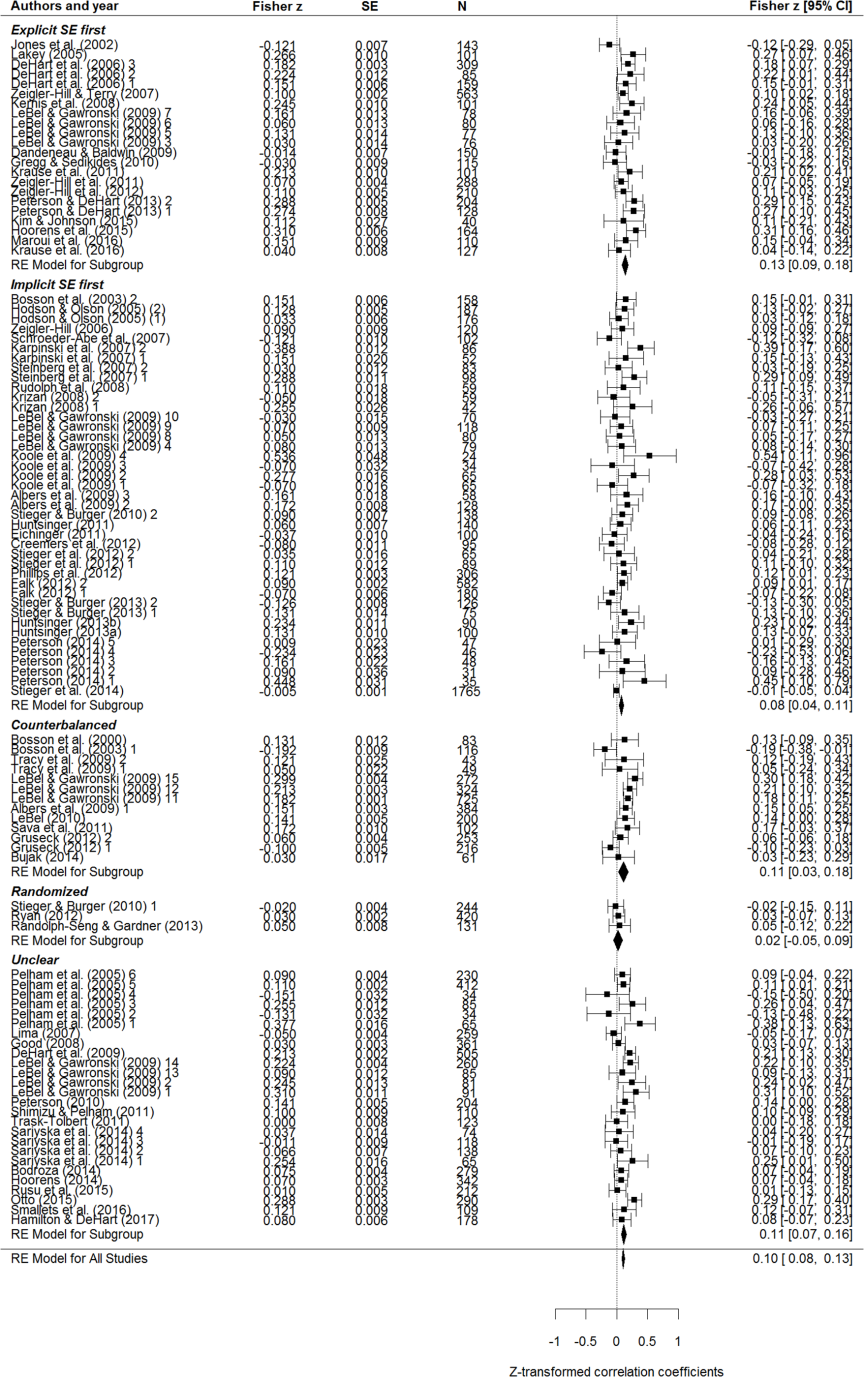
Forest plot of the associations between IPT-based implicit self-esteem and explicit self-esteem according to administration order.

Based on all included studies, we found a small-to-trivial positive correlation of *r* = .102 (95% CI: .079 to .125) between implicit and explicit self-esteem, indicating that the IPT shares about 1% of variance with explicit measures (the summary effect, sample effects, and subgroup effects according to administration order are detailed in Fig 2). As expected, correlations were somewhat stronger for published than for unpublished studies, although there was no significant difference between these two subgroups (Cochran’s *Q*(1) = 1.04, *p* = .309; see Table 1). However, overall and subgroup *I*^2^ values (i.e., the amount of true heterogeneity as opposed to heterogeneity due to sampling error and therefore chance) were small to medium-sized (according to established *I*^2^ values of classifications 0–25% suggest trivial, 25–50% small, 50–75% moderate, and 75–100% large heterogeneity; e.g., [42]), thus indicating a moderate amount of true cross-study effect heterogeneity which may be due to effect moderators.

**Table 1:** Associations of IPT scores with explicit self-esteem.

	*k*	*n*	*I*^2^	*r*	LCI	UCI	*p*
All studies	105	17777	50.83	.102	.079	.125	<.001
Published studies	78	13290	45.72	.110	.085	.135	<.001
Unpublished studies	27	4487	59.68	.081	.031	.131	.002

*Note*. *I*^2^ = percentage of between-effect variability because of true heterogeneity; LCI = lower bound of 95% confidence interval; UCI = upper bound of 95% confidence interval

Further subgroup analyses revealed no influence of administration order (i.e., implicit measure administered first vs. explicit measure administered first; *Q*(1) = 3.40, *p* = .065; see uppermost two blocks of Fig 2), administration type (pen-and-paper vs. computer administration; *Q*(1) = 2.426, *p* = .119), or rating type (liking vs. attractiveness of letters; *r*s = .089 and .139, respectively; *Q*(1) = 2.88, *p* = .090). Interestingly, IPT scores obtained by using the *B*-algorithm correlated significantly stronger with explicit self-esteem than IPT scores obtained with any other algorithm (*r*s = .13 and .06, respectively; Q(1) = 10.94, *p* < .001). Still, *I*^2^ values remained non-trivial (48.0% and 33.6%, respectively), thus indicating some extent of true between-study heterogeneity. Moreover, double administration of the IPT yielded weaker associations with explicit measures than single administration (*rs* = .02 and .11, respectively; *Q* (1) = 6.66, *p* = .010).

In the first block of a weighted stepwise hierarchical multiple meta-regression (Table 2), we first entered publication year as a single predictor which showed a negative sign of the regression coefficient, thus indicating decreasing effect sizes over time, although this failed to reach nominal significance. Adding the percentage of men within the samples as a predictor in a second step did not significantly contribute to variance explanation. Similarly, adding publication status to the model in a final step did not show significant influences of this predictor or improvements in model fit either. Examination of effect sizes according to the well-established benchmarks of Cohen [43] suggested a small influence of percentage of men indicating somewhat larger correlations between explicit and implicit measures for men, whilst the other predictors remained below the triviality threshold (i.e., η_p_^2^ < .02). However, because variance explanation of the models in all three steps of the regression analyses remained below the triviality threshold, our analysis indicates a lack of substantial influence of any of these three included predictors (see Table 2).

**Table 2:** Parameters of hierarchical linear weighted mixed-effects meta-regression on associations of implicit and explicit self-esteem measures.

Predictors	*b*	*SE*	β	*p*	η_p_^2^
First step					
	*k* = 105; *R*^2^ = <.001; *F*(1, 103) = 0.74				
Publication year	-0.001	0.004	-.002	.850	.001
Second step					
	*k* = 91; Δ*R*^2^ = <.001; *F*(2, 88) = 1.57				
Publication year	<0.001	0.004	.001	.952	<.001
Percentage of men in sample	-0.001	0.001	-.022	.117	.034
Final step					
	*k* = 91; Δ*R*^2^ = .022; *F*(3, 87) = 1.17				
Publication year	0.001	0.004	.002	.902	<.001
Percentage of men in sample	-0.001	0.001	-.021	.132	.032
Unpublished (0) vs. published (1)	0.020	0.031	.009	.508	.004

*Note*. Variables were weighted according to sample size; all *R*^2^ are adjusted values; *k* = number of samples; *b* = unstandardized regression coefficient; *SE* = standard error of unstandardized coefficient; β = standardized regression coefficient; changes in *R*^2^ between subsequent models were based on *k*s of higher order models; unpublished values include parameters that have been obtained through personal communications; all variance inflation factors (VIFs) < 1.10.

Sensitivity analyses did not show noteworthy effects of single studies on summary effect size estimates (*r*s ranging from .099 to .103). A similar stability of the overall evidence was observed when categorizing in regard to publication status (ranges of *r* in published vs. unpublished studies: .106 to .114, and .069 to .090).

Finally, we investigated correlations between first and last name IPT scores with explicit measures separately in a subset of our data, yielding trivial effects for first (*r* = .04, *p* = .100; 95% CI: -.008 to .096) and last name IPT scores (*r* = .02, *p* = .197; 95% CI: -.010 to .050). However, these summary effects were based on a comparatively small number of samples (*k* = 18).

### Dissemination bias

**Fig 3:**
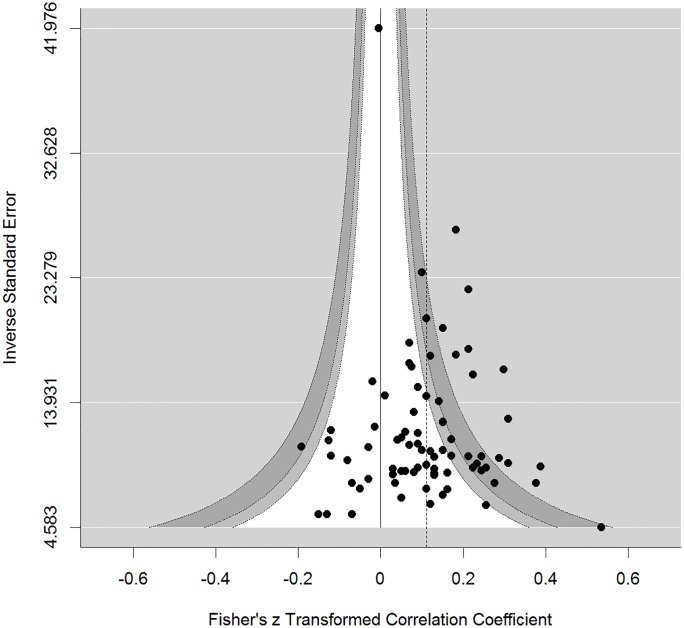
Contour-enhanced funnel plot for the *z*-transformed correlations between IPT-based implicit self-esteem and explicit self-esteem. Note: The vertical reference line represents the null effect; the confidence bands delimit non-significance of study effects inside (*p*s: white = .10, light grey = .05, dark grey = .01); the dashed vertical line represents the summary effect estimate.

Visual inspection of the funnel plot did not suggest evidence for publication bias in the present meta-analysis (Fig 3). Similarly, no evidence for bias was found by application of standard methods for publication bias detection, yielding non-significant results for the rank-order correlation method (*p* = .608), Sterne and Egger’s regression approach (*p* = .873), or the trim-and-fill method (0 studies added left of the estimated summary effect; no effect adjustment needed). Ioannidis and Trikalinos test of excess significance [37] did not show a nominally significant overrepresentation of significant hypothesis-conforming studies (*p* = .429), although more significant studies were observed than expected (24 observed vs. ~21 expected; the average analytic power of the primary studies included in the meta-analysis was 26.81%).

**Fig 4:**
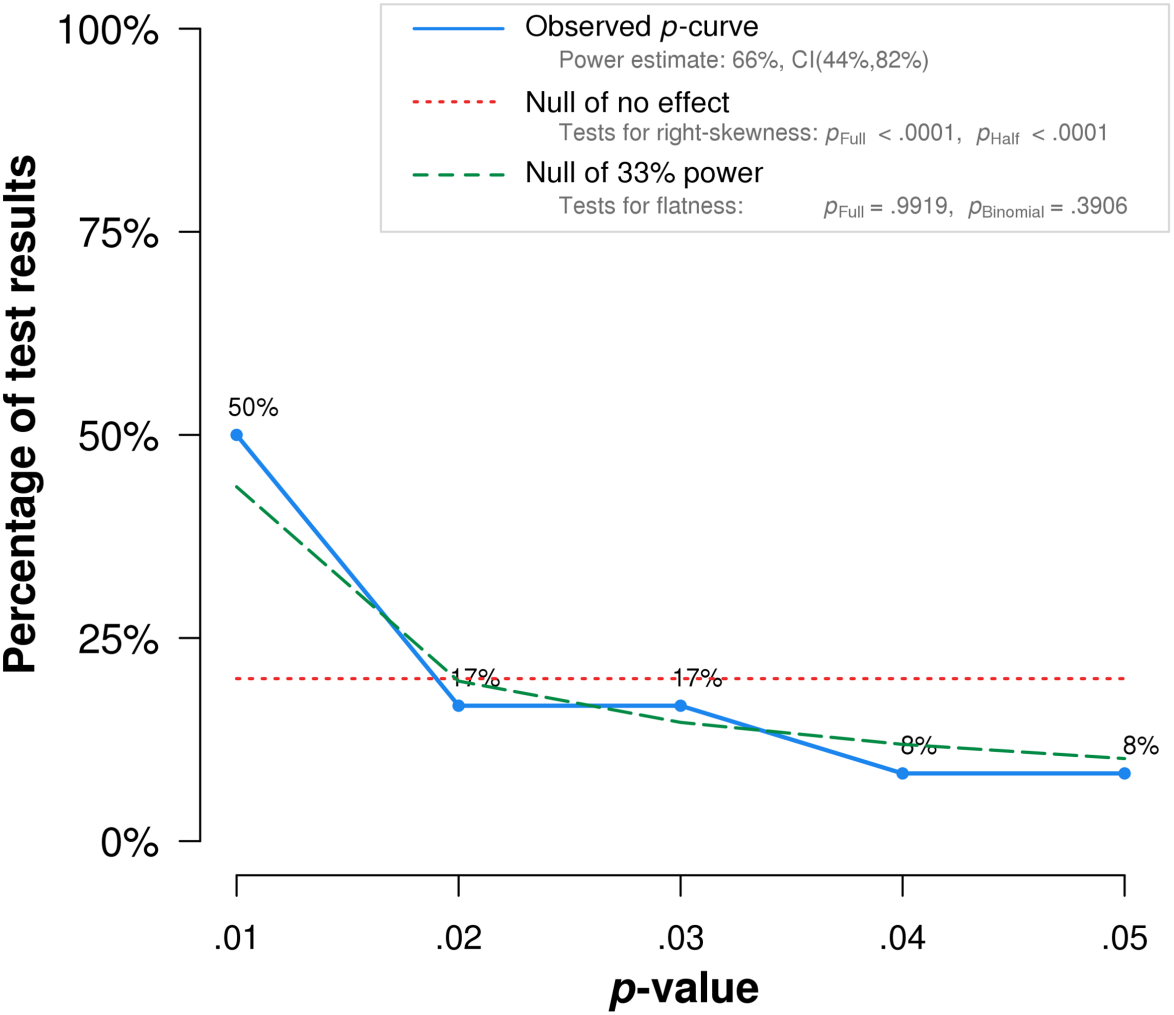
*p*-curve for significant positive studies. Note: The observed *p*-curve includes 24 statistically significant (*p* < .05) results, of which 16 are *p* < .025. There were 39 additional results entered but excluded from *p*-curve because they were *p* > .05.

Results from our *p*-curve analysis revealed no evidence for *p*-hacking, but showed that the available data provide evidential value (i.e., the *p*-curve is not flatter than a curve with an assumed power of 33%). However, the included significant positive studies on average were underpowered (average observed power = 66%; see Fig 4). The summary effect estimate estimated with the *p*-curve method (*r* = .163) was somewhat stronger than the conventional meta-analytic estimate, as based on all studies. Evidently, this is due to the omission of significant negative effects within the *p*-curve on the one hand, and the assumption of a fixed-effect model on the other hand.

For the *p*-uniform calculations, similar results were obtained. There was no evidence for *p*-hacking (*p* = .610), and the summary effect estimate was *r* = .154 (95% CI: .085 to .200). Again, the stronger summary effect, as compared with the conventionally calculated summary effect-size estimate, can be attributed to the necessary omission of all negative effect sizes that were nominally significant and the adoption of fixed-effect estimators.

The results from *p*-curve and *p*-uniform illustrate that these methods may be useful for the detection of several forms of dissemination bias, but might be less suitable and informative in the context of meta-analyses investigating small summary effects (i.e., when significant effects in both directions are likely to be encountered in the empirical literature). However, it should be noted that both methods performed comparatively well when comparing their effect estimates with conventional meta-analytic estimates, when only effect sizes with positive signs were included in the random-effects (*r* = .140) and fixed-effect calculations (*r* = .138).

## Discussion

In the present meta-analysis, we show, based on a large number of samples and participants, that implicit self-esteem, as measured with the IPT, is only marginally associated with explicit self-esteem (*r* = .102). This finding is consistent with an earlier meta-analytic estimate (*r* = .115; [14]), but contrasts these authors’ interpretation of a consistent and modest relationship between IPT-based implicit and explicit self-esteem [14]. Associations were small (if not trivial) at best and were mainly driven by IPT exposure and IPT algorithm type, as detailed below.

These findings can be interpreted in two ways. On the one hand, the lack of a noticeable association between implicit and explicit self-esteem could indicate that both constructs, conceptually or practically, are unrelated. This may mean that implicit and explicit self-esteem are orthogonal dimensions that represent different constructs, a notion that already has received some support in the literature (e.g., [16]) and is largely in line with expectations of the dual-process models (e.g, [10]). This idea is supported by the observation that implicit and explicit self-esteem measures predict different behaviors and traits. For instance, implicit self-esteem has been shown to be related to non-verbal anxiety signs, higher levels of internet addiction, higher romantic jealousy in men, but not to be significantly related to depression scores, whilst explicit self-esteem has been related to self-rated anxiety, lower levels of internet addiction, lower romantic jealousy in women, and lower depression scores [44–47]. Consequently, the validity of the IPT is difficult to determine (e.g., [18]). Therefore, it may also be argued that this interpretation calls the use of the term “implicit self-esteem” into question because even for dual-process models a certain association between implicit and explicit evaluations would be expected [28]. Consequently, initial letter preferences may perhaps be better termed “implicit name letter evaluations”.

On the other hand, our findings may indicate that the IPT is an unsuitable measure for the assessment of implicit self-esteem which appears to be supported by evidence from recent studies (e.g., [15]). This concern is exacerbated by the observation that the reliability of the IPT has typically been observed to be meagre (i.e., unacceptably low internal consistency figures, ranging from the low .30s to the low .50s, depending on the algorithm used; [17]; and low retest reliabilities, ranging from the high .30s to the high .60s; [16,48,49]). However, it should be noted that for the IPT, the observed retest reliabilities necessarily are based on two items only which, to a certain extent, would account for these suboptimal characteristics.

It has been argued that one explanation for small correlations between IPT-based implicit self-esteem and explicit self-esteem measures may be due to the low reliabilities of the IPT, which invariably must limit the observable strength of the correlation coefficient. Other causes, such as unique method variance or differences in conceptual approaches between explicit and implicit measures have also been cited as potential causes for such small correlations [50,51].

In this vein, it seems interesting that correlations between the IPT and explicit self-esteem measures were higher for the *B*-algorithm than for any other algorithm used. Because the *B*-algorithm does not control for individual response tendencies that are controlled for in other algorithms (see [17]), this may mean that mere response tendencies (e.g., acquiescence) may well be genuinely related to explicit self-esteem.

In a different vein, stronger correlations of single than double administrations of the IPT with explicit measures may be due to the already mentioned low retest reliability of the IPT on the one hand (which makes it more difficult to detect true effects), but also to responders increasingly recognizing the intended purpose of the IPT, on the other hand. This finding is consistent with those of a previous study [52]. The former potential cause reflects a general psychometric issue of the IPT, although it should be noted that double administration-based reliabilities have been shown to be preferable to single administrations in some studies (e.g., [49]). The latter cause may be attributed to an ever-increasing number of participants who recognize the implicit purpose of the IPT on the second administration (i.e., reflecting implicit theories about the purpose of name letter ratings, as already demonstrated in previous studies; see, [53]).

Another meaningful factor may be that indirect measures such as the IPT may tap into states rather than traits (e.g., [54]). This may be responsible for the reported low retest reliabilities. The above points support the idea, that the IPT is a useful measure for a certain construct, however, this construct still remains to be clarified.

Previously reported differences of administration order [14], indicating higher correlations when explicit measures were administered first than when implicit measures were administered first, did not emerge in the present meta-analysis. Indeed, the summary subgroup effect of samples where explicit measures were administered first was numerically larger. However, this effect did not significantly differ from administrations where these measures were presented in the opposite order (confidence intervals overlapped considerably). This sheds further doubt on the interpretation of IPT scores as expressions of self-esteem, because the heightened accessibility of self-esteem due to the initial administration of an explicit self-esteem measure should lead to increases of the association between explicit and implicit measures (see, [16]).

Interestingly, effect sizes were somewhat (albeit non-significantly) stronger for ratings of letter attractiveness than for letter liking. This finding is consistent with the idea that liking and attractiveness evaluations may reflect different domains of implicit self-esteem [55].

As expected, the regression coefficients indicated decreasing strengths of correlations over time (see, [13]) although the sign changed when additional moderators where included in the regression model and unpublished studies yielded (non-significantly) smaller summary effect sizes than published studies. Consistent with these results, the methods testing for dissemination bias we applied did not yield evidence for publication and reporting bias or *p*-hacking.

Although the above discussed causes are likely candidates to account for the small-to-trivial correlation between the IPT and explicit self-esteem scores, the implications for the use of the IPT (at least as a measure for implicit self-esteem) are somewhat disheartening. The suboptimal IPT stabilities and its conceptual ambiguity limit the evidential value that can be derived from IPT-based assessments.

### Limitations

Of course, a more detailed assessment of differences between the use of specific algorithms for implicit self-esteem score calculations would have been desirable. However, the comparatively low application numbers of non-*B*-algorithms did not allow for meaningful comparisons of the *B*-algorithm with other calculation methods. Still, the present investigation suggests that the *B*-algorithm yields scores that might be more meaningfully associated with explicit self-esteem than scores from other popularized algorithms.

Some of the available studies used a compound measure of birthday number liking and the IPT as an indirect measure (*k* = 5) which may impair comparability between the observed effects. However, there was no significant difference between subgroup summary effects (*p* > .05).

Moreover, our estimates of first and last initial-only correlations with explicit self-esteem were based on a comparatively small number of samples. However, the effect emerged in the expected direction, yielding numerically stronger estimates for first than for last initials (i.e., consistent with observations that first initial name letter ratings are more meaningful than last name letter ratings; [11]), although confidence intervals of effect estimates overlapped and the effects did not reach nominal significance.

Another point that needs to be noted is that the observed between-study heterogeneity limits the validity of *p*-curve- and *p*-uniform-based effect estimates. Both methods have been shown to overestimate effect sizes in presence of moderate between-study heterogeneity [25], which may explain the stronger effect estimates of both methods than in our conventional analysis. Another point that likely contributed to effect overestimation is that *p*-values that are associated with hypothesis non-conforming effect directions (i.e., negative signs in the present meta-analysis) cannot be included in *p*-curve or *p*-uniform effect estimations, thus necessarily causing effect inflation of summary effects.

It would have been desirable to compare the results of our meta-analysis with the results of the samples that had been included in Krizan and Suls’ study [14]. However, unfortunately these primary study details have not been documented in the previous meta-analytical account.

### Future directions

Based on the present findings, the validity of implicit self-esteem as measured by the IPT appears to be unclear. This may be partly due to the fact that many of the recommendations pertaining to the administration of the IPT (e.g., [11,12,14]) have not received sufficient attention in the subsequent studies. Apparently, the originally suggested administration procedure [2] seems to be the most frequently used design, whilst variations in procedural characteristics have been infrequently implemented. In particular, the identified moderating factors of IPT and explicit self-esteem associations, such as algorithm type, IPT exposure (single vs. double administration), or rating type (liking vs. attractiveness) need further attention to identify the meaning and nature of implicit self-esteem.

### Concluding remarks

In all, we show that there is no noteworthy association between IPT-based implicit and explicit self-esteem. These findings broadly support dual-process models of implicit and explicit evaluations but may also be due to suboptimal psychometric properties of the IPT on the one hand, and lacking validity of the IPT on the other hand. So far, the latent construct as measured by the IPT remains unclear. Further examination of IPT-based correlates with behavioral data may shed light on the meaning and nature of IPT scores.

## Supporting information

S1 Text References of included and excluded studies. (DOC)

S2 Text R-code for included analyses. (DOCX)

S1 Table Characteristics of included samples. (DOCX)

S2 Table PRISMA checklist. (DOC)
