# Supplementary material for: Indirect (implicit) and direct (explicit) self-esteem measures are virtually unrelated: A meta-analysis of the initial preference task
Source: PLoS One. 2018 Sep 6;13(9):e0202873. doi: 10.1371/journal.pone.0202873 (PMC6126831; doi:10.1371/journal.pone.0202873)
Supplement: S1 Text — (DOC) [file pone.0202873.s001.doc]

**Included studies**

Albers, L., Rotteveel, M., & Dijksterhuis, A. P. (2009). Towards optimizing the lame letter test as a measure of implicit self-esteem. *Self and Identity*, *8*, 63-77. http://doi.org/10.1080/15298860802091062

Bodroza, B. (2014). Validation of two conceptualizations of fragile self-esteem: Contingent high self-esteem and incongruent high self-esteem. *Psihologija*, *47*, 373-391. <http://doi.org/10.2298/PSI1404373B>

Bosson, J. K., Brown, R. P., Zeigler-Hill, V., & Swann, W. B. (2003). Self-enhancement tendencies among people with high explicit self-esteem: The moderating role of implicit self-esteem. *Self and Identity*, *2*, 169-187. <http://doi.org/10.1080/15298860390208801>

Bosson, J. K., Swann, W. B., & Pennebaker, J. W. (2000). Stalking the perfect measure of implicit self-esteem: The blind men and the elephant revisited? *Journal of Personality and Social Psychology*, *79*, 631-643. <http://doi.org/10.1037//0022-3514.79.4.631>

Bujak, D. E. (2014). *A test of the implicit egotism explanation for the name-letter effect*. Unpublished master’s thesis, Central Connecticut State University, CT.

Creemers, D. H. M., Scholte, R. H. J., Engels, R. C. M. E., Prinstein, M. J., & Wiers, R. W. (2012). Implicit and explicit self-esteem as concurrent predictors of suicidal ideation, depressive symptoms, and loneliness. *Journal of Behavior Therapy and Experimental Psychiatry*, *43*, 638-646. <http://doi.org/10.1016/j.jbtep.2011.09.006>

Dandeneau, S. D., & Baldwin, M. W. (2009). The buffering effects of rejection-inhibiting attentional training on social and performance threat among adult students. *Contemporary Educational Psychology*, *34*, 42-50. http://doi.org/10.1016/j.cedpsych.2008.05.004

DeHart, T., Pelham, B. W., & Tennen, H. (2006). What lies beneath: Parenting style and implicit self-esteem. *Journal of Experimental Social Psychology*, *42*, 1-17. <http://doi.org/10.1016/j.jesp.2004.12.005>

DeHart, T., Tennen, H., Armeli, S., Todd, M., & Mohr, C. (2009). A diary study of implicit self-esteem, interpersonal interactions and alcohol consumption in college students. *Journal of Experimental Social Psychology, 45*, 720-730.

Eichinger, T. (2011). *Wovon man nicht sprechen kann, darüber muss man schweigen? Validität eines neuen impliziten Maßes zur Messung von Selbstwert: Self-esteem affect misattribuation procedure*. Unpublished master’s thesis, University of Vienna, Austria.

Falk, C. F. (2012). *Is there cultural variability in implicit self-esteem?* Unpublished doctoral dissertation, University of British Columbia, Vancouver, BC.

Good J. J. (2008). *Reconciling the costs and benefits of gender conformity: The role of motivation*. Unpublished master’s thesis, State University of New Jersey, NJ.

Gregg, A. P., & Sedikides, C. (2010). Narcissistic fragility: Rethinking its links to explicit and implicit self-esteem. *Self and Identity, 9*, 142-161.

Gruseck, D. J. (2012). Der Zusammenhang von implizitem und explizitem Selbstwert mit Stress: Ein Vergleich zwischen österreichischen und spanischen Studierenden. *Unpublished master’s thesis, University of Vienna, Austria.*

Gschwendner, T., Hofmann, W., & Schmitt, M. (2006). Moderators of the consistency of implicitly and explicitly assessed attitudes and personality traits. *Psychologische Rundschau*, *57*, 13-33. http://doi.org/10.1026/0033-3042.57.1.13

Hamilton, H. R., & DeHart, T. (2017). Drinking to belong: The effect of a friendship threat and self-esteem on college student drinking. *Self & Identity, 16*, 1-15.

Hodson, G., & Olson, J. M. (2005). Testing the generality of the name letter effect: Name initials and everyday attitudes. *Personality and Social Psychology Bulletin*, *31*, 1099-1111. <http://doi.org/10.1177/0146167205274895> (Personal communication: G. Hodson to J.P., July 17, 2018).

Hoorens, V. (2014). What’s really in a Name-Letter Effect? Name-letter preferences as indirect measures of self-esteem. *European Review of Social Psychology*, *25*, 228-262. http://doi.org/10.1080/10463283.2014.980085

Hoorens, V., Takano, K., Franck, E., Roberts, J. E., & Raes, F. (2015). Initial and noninitial name-letter preferences as obtained through repeated letter rating tasks continue to reflect (different aspects of) self-esteem. *Psychological Assessment*, *27*, 905-914. <http://doi.org/10.1037/pas0000092>

Huntsinger, J. R. (2011). Mood and trust in intuition interactively orchestrate correspondence between implicit and explicit attitudes. *Personality and Social Psychology Bulletin*, *37*, 1245-1258. http://doi.org/10.1177/0146167211410069

Huntsinger, J. R. (2013a). Anger enhances correspondence between implicit and explicit attitudes. *Emotion*, *13*, 350-357. http://doi.org/10.1037/a0029974

Huntsinger, J. R. (2013b). Narrowing down to the automatically activated attitude: A narrowed conceptual scope improves correspondence between implicitly and explicitly measured attitudes. *Journal of Experimental Social Psychology*, *49*, 132-137. http://doi.org/10.1016/j.jesp.2012.07.018

Jones, J. T., Pelham, B. W., Mirenberg, M. C., & Hetts, J. J. (2002). Name letter preferences are not merely mere exposure: Implicit egotism as self-regulation. *Journal of Experimental Social Psychology, 38*, 170-177.

Karpinski, A., Steinberg, J. A., Versek, B., & Alloy, L. B. (2007). The Breadth-based Adjective Rating Task (BART) as an indirect measure of self-esteem. *Social Cognition*, *25*, 778-818. <http://doi.org/10.1521/soco.2007.25.6.778>

Kernis, M. H., Lakey, C. E., & Heppner, W. L. (2008). Secure versus fragile high self-esteem as a predictor of verbal defensiveness: Converging findings across three different markers. *Journal of Personality, 76*, 477-512.

Kim, K., & Johnson, M. K. (2015). Distinct neural networks support the mere ownership effect under different motivational contexts. *Social Neuroscience*, *10*, 376-390. <http://doi.org/10.1080/17470919.2014.999870> (Personal communication: K. Kim to J.P., July 31, 2018).

Koole, S. L., Govorun, O., Cheng, C. M., & Gallucci, M. (2009). Pulling yourself together: Meditation promotes congruence between implicit and explicit self-esteem. *Journal of Experimental Social Psychology*, *45*, 1220-1226. http://doi.org/10.1016/j.jesp.2009.05.018

Krause, S., Back, M. D., Egloff, B., & Schmukle, S. C. (2011). Reliability of implicit self-esteem measures revisited. *European Journal of Personality*, *25*, 239-251. <http://doi.org/10.1002/per.792>

Krause, S., Back, M. D., Egloff, B., & Schmukle, S. C. (2016). Predicting self-confident behaviour with implicit and explicit self-esteem measures. *European Journal of Personality, 30*, 648-662.

Krizan, Z. (2008). What is implicit about implicit self-esteem? *Journal of Research in Personality*, *42*, 1635-1640. <http://doi.org/10.1016/j.jrp.2008.07.002>

Lakey, C. E. (2003). *Secure versus fragile high self-esteem and verbal self-esteem*. Unpublished master’s thesis, Western Carolina University, NC.

Lebel, E. P. (2010). Attitude accessibility as a moderator of implicit and explicit self-esteem correspondence. *Self and Identity*, *9*, 195-208. <http://doi.org/10.1080/15298860902979166>

LeBel, E. P., & Gawronski, B. (2009). How to find what’s in a name: Scrutinizing the optimality of five scoring algorithms for the name-letter task. *European Journal of Personality*, *23*, 85-106. http://doi.org/10.1002/per.705

Lima, E. N. (2007). *The association between narcissism and implicit self-esteem: A test of the fragile self-esteem hypothesis*. Unpublished master’s thesis, Florida State University, FL.

Maroui, C., Maricutoiu, L. P., & Sava, F. A. (2016). Explicit self-esteem and contingencies of self-worth: The moderating role of implicit self-esteem. *Personality and Individual Differences, 99*, 235-241.

Pelham, B. W., Koole, S. L., Hardin, C. D., Hetts, J. J., Seah, E., & DeHart, T. (2005). Gender moderates the relation between implicit and explicit self-esteem. *Journal of Experimental Social Psychology*, *41*, 84-89. http://doi.org/10.1016/j.jesp.2003.10.008

Peterson, J. L. (2010). *I love you (but I can't look you in the eyes): Explicit and implicit self-esteem predict verbal and nonverbal response to relationship threat*. Unpublished master’s thesis, Loyola University Chicago, IL.

Peterson, J. L. (2014). Explicit thoughts of security activate implicit self-doubt in anxiously attached participants. *Personal Relationships*, *21*, 206-224. <http://doi.org/10.1111/pere.12027> (Personal communication: J. L. Peterson to J.P., July 17, 2018).

Peterson, J. L., & DeHart, T. (2013). Regulating connection: Implicit self-esteem predicts positive non-verbal behavior during romantic relationship-threat. *Journal of Experimental Social Psychology, 49*, 99-105. [http://dx.doi.org/10.1016/j.jesp.2012.07.013](http://psycnet.apa.org/doi/10.1016/j.jesp.2012.07.013) (Personal communication: J. L. Peterson to J.P., July 24, 2018).

Phillips, W. J., Hine, D. W., & Bhullar, N. (2012). A latent profile analysis of implicit and explicit cognitions associated with depression. *Cognitive Therapy and Research*, *36*, 458-473. <http://doi.org/10.1007/s10608-011-9381-z>

Randolph-Seng, B., & Gardner, W. L. (2013). Validating measures of leader authenticity: Relationships between implicit/explicit self-esteem, situational cues, and leader authenticity. *Journal of Leadership & Organizational Studies, 20*, 214-231.

Rudolph, A., Schröder-Abe, M., Schütz, A., Gregg, A. P., & Sedikides, C. (2008). Through a glass, less darkly? Reassessing convergent and discriminant validity in measures of implicit self-esteem. *European Journal of Psychological Assessment, 24*, 273-281.

Rusu, A., Mairean, C., Hojbota, A.-M., Gherasim, L. R., & Gavriloaiei, S. I. (2015). Relationships of career adaptabilities with explicit and implicit self-concepts. *Journal of Vocational Behavior*, *89*, 92-101. http://doi.org/10.1016/j.jvb.2015.05.003

Ryan, J. F. (2012). *You had me at Helen: The name letter effect in judgements of humor*. Unpublished master’s thesis, Loyola University Chicago, IL.

Sariyska, R., Reuter, M., Bey, K., Sha, P., Li, M., Chen, Y.-F., … Montag, C. (2014). Self-esteem, personality and internet addiction: A cross-cultural comparison study. *Personality and Individual Differences*, *61-62*, 28-33. <http://doi.org/10.1016/j.paid.2014.01.001> (Personal communication: R. Sariyska to J.P., July 27, 2018).

Sava, F. A., Maricutoiu, L. P., Rusu, S., Macsinga, I., & Virga, D. (2011). Implicit and explicit self-esteem and irrational beliefs. *Journal of Cognitive and Behavioral Psychotherapies, 11*, 97-111.

Schroeder-Abe, M., Rudolph, A., Wiesner, A., & Schuetz, A. (2007). Self-esteem discrepancies and defensive reactions to social feedback. *International Journal of Psychology*, *42*, 174-183. http://doi.org/10.1080/00207590601068134

Shimizu, M., & Pelham, B. W. (2011). Liking for positive words and icons moderates the association between implicit and explicit self-esteem. *Journal of Experimental Social Psychology, 47*, 994-999.

Smallets, S., Streamer, L., Kondrak, C. L., & Seery, M. D. (2016). Bringing you down versus bringing me up: Discrepant versus congruent high explicit self-esteem differentially predict malicious and benign envy. *Personality and Individual Differences, 94*, 173-179.

Steinberg, J. A., Karpinski, A., & Alloy, L. B. (2007). The exploration of implicit aspects of self-esteem in vulnerability—stress models of depression. *Self and Identity*, *6*, 101-117. <http://doi.org/10.1080/15298860601118884>

Stieger, S., & Burger, C. (2010). Implicit and explicit self-esteem in the context of internet addiction. *Cyberpsychology Behavior and Social Networking*, *13*, 681-688. <http://doi.org/10.1089/cyber.2009.0426>

Stieger, S., & Burger, C. (2013). More complex than previously thought: New insights into the optimal administration of the initial preference task. *Self and Identity*, *12*, 201-216. <http://doi.org/10.1080/15298868.2012.655897>

Stieger, S., Preyss, A. V., & Voracek, M. (2012). Romantic jealousy and implicit and explicit self-esteem. *Personality and Individual Differences*, *52*, 51-55. http://doi.org/10.1016/j.paid.2011.08.028

Stieger, S., Voracek, M., & Nader, I. W. (2014). Parent-child proximity: automatic cognitions matter. *Social Indicators Research*, *119*, 967-978. http://doi.org/10.1007/s11205-013-0524-3

Tracy, J. L., Cheng, J. T., Robins, R. W., & Trzesniewski, K. H. (2009). Authentic and hubristic pride: The affective core of self-esteem and narcissism. *Self and Identity, 8*, 169-213.

Trask-Tolbert, A. R. (2011). *Explaining variance in counterfactual-seeking behavior*. Unpublished master’s thesis, Miami University, Oxford, OH.

Zeigler-Hill, V. (2006). Discrepancies between implicit and explicit self-esteem: Implications for narcissism and self-esteem instability. *Journal of Personality*, *74*, 119-143. http://doi.org/10.1111/j.1467-6494.2005.00371.x

Zeigler-Hill, V., & Terry, C. (2007). Perfectionism and explicit self-esteem: The moderating role of implicit self-esteem. *Self and Identity*, *6*, 137-153. <http://doi.org/10.1080/15298860601118850>

Zeigler-Hill, V., Clark, C. B., & Beckman, T. E. (2011). Fragile self-esteem and the interpersonal circumplex: Are feelings of self-worth associated with interpersonal style? *Self and Identity*, *10*, 509-536. http://doi.org/10.1080/15298868.2010.497376

Zeigler-Hill, V., Fulton, J. J., & McLemore, C. (2012). Discrepancies between explicit and implicit self-esteem: Implications for mate retention strategies and perceived infidelity. *Journal of Social Psychology*, *152*, 670-686. http://doi.org/10.1080/00224545.2012.688778

**No primary data**

Alicke, M. D., & Sedikides, C. (2009). Self-enhancement and self-protection: What they are and what they do. *European Review of Social Psychology*, *20*, 1-48. <http://doi.org/10.1080/10463280802613866>

Arndal, M. F., Kjaer, F., Elling, E. J., Brandt, K. M., & Juncker-Londal, T. (2015). *Velfaerdsstatens patologi*. Unpublished bachelor’s thesis, Roskilde University, Denmark.

Banse, R., & Greenwald, A. G. (2007). Personality and implicit social cognition research: Past, present and future. *European Journal of Personality*, *21*, 371-382. http://doi.org/10.1002/per.638

Bargh, J. A., Schwader, K. L., Hailey, S. E., Dyer, R. L., & Boothby, E. J. (2012). Automaticity in social-cognitive processes. *Trends in Cognitive Sciences*, *16*, 593-605. http://doi.org/10.1016/j.tics.2012.10.002

Bertamini, M., & Casati, R. (2009). False beliefs and naive beliefs: They can be good for you. *Behavioral and Brain Sciences*, *32*, 512-513. <http://doi.org/10.1017/S0140525X09991178>

Bohner, G., & Waenke, M. (2009). The psychology of attitudes and persuasion. In J. L. Wood & T. A. Gannon (eds.), *Public opinion and criminal justice* (pp. 3-32). Cullompton, UK: Willan.

Bosson, J. & Swann, W. B., Jr. (2009). Self-esteem: Nature, origins, and consequences. In R. Hoyle & M. Leary (eds.), *Handbook of individual differences in social behavior* (pp. 527-546). New York: Guilford.

Brown, R. P., & Bosson, J. K. (2001). Narcissus meets Sisyphus: Self-love, self-loathing, and the never-ending pursuit of self-worth. *Psychological Inquiry*, *12*, 210-213.

Buhrmester, M. D., Blanton, H., & Swann, W. B., Jr. (2011). Implicit self-esteem: Nature, measurement, and a new way forward. *Journal of Personality and Social Psychology, 100*, 365-385.

Burke, B. L., Martens, A., & Faucher, E. H. (2010). Two decades of terror management theory: A meta-analysis of mortality salience research. *Personality and Social Psychology Review*, *14*, 155-195. http://doi.org/10.1177/1088868309352321

De Raedt, R. (2006). Activation of implicit self-schemas and a difficulty to disengage from negative cognitions in depression: An experimental psychopathology approach. *Psychologica Belgica*, *46*, 117-130.

Devos, T. (2008). Implicit attitudes 101: Theoretical and empirical insights. In W. D. Crano & R. Prislin (eds.), *Attitudes and attitude change* (pp. 61-84). New York: Psychology Press.

Devos, T., & Banaji, M. R. (2003). Implicit self and identity. In J. LeDoux, J. Debiec, & H. Moss (eds.), *Self: From soul to brain* (pp. 177-211). New York: New York Academy of Sciences.

Donnellan, M. B., Trzesniewski, K. H., & Robins, R. W. (2015). *Measures of self-esteem*. In G. J. Boyle, D. H. Saklofske, & G. Matthews (eds.), *Measures of personality and social psychological constructs* (pp. 131-157). New York: Academic Press.

Doris, J. M. (2009). Skepticism about persons. *Philosophical Issues, 19*, 57-91.

Dunning, D. (2007). Self-image motives and consumer behavior: how sacrosanct self-beliefs sway preferences in the marketplace. *Journal of Consumer Psychology*, *17*, 237-249. http://doi.org/10.1016/S1057-7408(07)70033-5

Dyjas, O., Grasman, R. P. P. P., Wetzels, R., Van der Maas, H. L. J., & Wagenmakers, E.-J. (2012). What’s in a name: A Bayesian hierarchical analysis of the name-letter effect. *Frontiers in Psychology*, *3*, 334. http://doi.org/10.3389/fpsyg.2012.00334

Falk, C. F., & Heine, S. J. (2015). What is implicit self-esteem, and does it vary across cultures? *Personality and Social Psychology Review, 19*, 177-198.

Fazio, R. H., & Olson, M. A. (2003). Implicit measures in social cognition research: Their meaning and use. *Annual Review of Psychology*, *54*, 297-327. http://doi.org/10.1146/annurev.psych.54.101601.145225

Gallucci, M. (2003). I sell seashells by the seashore and my name is Jack: Comment on Pelham, Mirenberg, and Jones (2002). *Journal of Personality and Social Psychology*, *85*, 789-799. http://doi.org/10.1037/0022-3514.85.5.789

Gelitz, C. (2009). Die Macht der Initialen. *Gehirn & Geist, 3*, 18-23.

Gilson, L. L., Litchfield, R. C., & Gilson, P. W. (2014). An examination of the relationship between time and creativity: Applying a temporal lens to the study of creativity. In A. J. Shipp & Y. Fried (eds.), *Time and work: How time impacts individuals* (pp. 141-162). New York: Taylor & Francis.

Gore, J. S., & Cross, S. E. (2014). Who am I becoming? A theoretical framework for understanding self-concept change. *Self and Identity*, *13*, 740-764. http://doi.org/10.1080/15298868.2014.933712

Greenwald, A. G. (1990). What cognitive representations underlie social-attitudes. *Bulletin of the Psychonomic Society*, *28*, 254-260.

Greenwald, A. G., & Banaji, M. R. (1995). Implicit social cognition: Attitudes, self-esteem, and stereotypes. *Psychological Review*, *102*, 4-27. http://doi.org/10.1037//0033-295X.102.1.4

Greenwald, A. G., & Farnham, S. D. (2000). Using the implicit association test to measure self-esteem and self-concept. *Journal of Personality and Social Psychology*, *79*, 1022-1038. http://doi.org/10.1037//0022-3514.79.6.1022

Gregg, A. P., & Mahadevan, N. (2014). Intellectual arrogance and intellectual humility: An evolutionary-epistemological account. *Journal of Psychology and Theology*, *42*, 7-18.

Gronas, M. (2009). Just what word did Mandel’shtam forget? A mnemopoetic solution to the problem of Saussure’s anagrams. *Poetics Today*, *30*, 155-205. http://doi.org/10.1215/03335372-2008-007

Gronas, M. (2011). *Cognitive poetics and cultural memory: Russian literary mnemonics* (Vol. 28). New York: Routledge.

Haines, E. L., & Sumner, K. E. (2013). Digging deeper or piling it higher? Implicit measurement in organizational behavior and human resource management. *Human Resource Management Review*, *23*, 229-241. http://doi.org/10.1016/j.hrmr.2012.12.004

Haoming, L., & Jijia, Z. (2004). Research and development on the structure of self-esteem. *Advances in Psychological Science*, *12*, 567-572.

Hofmann, W., Gawronski, B., Gschwendner, T., Le, H., & Schmitt, M. (2005). A meta-analysis on the correlation between the implicit association test and explicit self-report measures. *Personality and Social Psychology Bulletin, 31*, 1369-1385.

Hoorens, V. (1990). Nuttin’s affective selfparticles hypothesis and the name letter effect: A review. *Psychologica Belgica*, *30*, 23-48.

Hoorens, V., & Desrichard, O. (2002). Self-other asymmetries: Three wonders in “hot” social cognition and three questions about them. *Psychologica Belgica*, *42*, 3-21.

Igou, E. R., & van Tilburg, W. A. P. (2015). Ahead of others in the authorship order: names with middle initials appear earlier in author lists of academic articles in psychology. *Frontiers in Psychology*, *6*, 469. http://doi.org/10.3389/fpsyg.2015.00469

Jordan, C. H., & Zeigler-Hill, V. (2013). Fragile self-esteem: The perils and pitfalls of (some) high self-esteem. In V. Zeigler-Hill (ed.), *Self-esteem* (pp. 80-98). New York: Psychology Press.

Kernis, M. H. (2003). Toward a conceptualization of optimal self-esteem. *Psychological Inquiry*, *14*, 1-26. <http://doi.org/10.1207/S15327965PLI1401_01>

Koole, S. L., & DeHart, T. (2007). Self-affection without self-reflection: Origins, models, and consequences of implicit self-esteem. In Spencer, S. J. (ed.), *The self: Frontiers of social psychology* (pp. 21-49). New York: Psychology Press.

Koole, S. L., & Pelham, B. W. (2003). On the nature of implicit self-esteem: The case of the name letter effect. In S. J. Spencer, S. Fein, M. P. Zanna, & J. M. Olson, (eds.), *Motivated social perception: The Ontario Symposium* (pp. 93-116). Mahwah, NJ: Lawrence Earlbaum.

Krizan, Z., & Suls, J. (2008). Are implicit and explicit measures of self-esteem related? A meta-analysis for the Name-Letter Test. *Personality and Individual Differences*, *44*, 521-531. <http://doi.org/10.1016/j.paid.2007.09.017>

LeBel, E. P., & Paunonen, S. V. (2011). Sexy but often unreliable: The impact of unreliability on the replicability of experimental findings with implicit measures. *Personality and Social Psychology Bulletin*, *37*, 570-583. http://doi.org/10.1177/0146167211400619

Kwan, V. S. Y., & Mandisodza, A. N. (2007). Self-esteem: On the relation between conceptualization and measurement. In C. Sedikides & S. J. Spencer (eds.), *The self* (pp. 259-282). New York: Psychology Press.

Markus, H., & Wurf, E. (1987). The dynamic self-concept: A social psychological perspective. *Annual Review of Psychology*, *38*, 299-337. http://doi.org/10.1146/annurev.psych.38.1.299

Meyers, C. D. (2015). Automatic behavior and moral agency: Defending the concept of personhood from empirically based skepticism. *Acta Analytica-International Periodical for Philosophy in the Analytical Tradition*, *30*, 193-209. http://doi.org/10.1007/s12136-014-0239-3

Miller, J. M., & Krosnick, J. A. (1998). The impact of candidate name order on election outcomes. *Public Opinion Quarterly*, *62*, 291-330. http://doi.org/10.1086/297848

Mitchell, M. L., & Jolley, J. M. (1999). The correlator: A self-guided tutorial. *Teaching of Psychology*, *26*, 298-299. <http://doi.org/10.1207/S15328023TOP260410>

Moreland, R. L., & Topolinski, S. (2010). The mere exposure phenomenon: A lingering melody by Robert Zajonc. *Emotion Review*, *2*, 329-339. <http://doi.org/10.1177/1754073910375479>

Moss, S. A., Dowling, N., & Callanan, J. (2009). Towards an integrated model of leadership and self regulation. *Leadership Quarterly*, *20*, 162-176. http://doi.org/10.1016/j.leaqua.2009.01.005

Nosek, B. A., Hawkins, C. B., & Frazier, R. S. (2011). Implicit social cognition: From measures to mechanisms. *Trends in Cognitive Sciences*, *15*, 152-159. <http://doi.org/10.1016/j.tics.2011.01.005>

Otto, B. (2015). *The relation between implicit and explicit self-esteem predicting inconsistent parenting*. Unpublished master’s thesis, Loyola University Chicago, IL.

Pannese, A. (2011). The “I” of the beholder: studying the “self” across the humanities and neuroscience. *Medical Humanities*, *37*, 115-122. http://doi.org/10.1136/jmh.2011.007369

Pelham, B. W., Carvallo, A., & Jones, J. T. (2005). Implicit egotism. *Current Directions in Psychological Science*, *14*, 106-110. http://doi.org/10.1111/j.0963-7214.2005.00344.x

Pelham, B. W., Mirenberg, M. C., & Jones, J. T. (2002). Why Susie sells seashells by the seashore: Implicit egotism and major life decisions. *Journal of Personality and Social Psychology*, *82*, 469-487. <http://doi.org/10.1037//0022-3514.82.4.469>

Phillips, W. J., Hine, D. W., & Thorsteinsson, E. B. (2010). Implicit cognition and depression: A meta-analysis. *Clinical Psychology Review*, *30*, 691-709. <http://doi.org/10.1016/j.cpr.2010.05.002>

Sedikides, C., Gregg, A. P., & Hart, C. M. (2007). The importance of being modest. In C. Sedikides & S. J. Spencer (eds.), *The self* (pp. 163-184). New York: Psychology Press.

Spitzer, M. (2006). Names: Nothing like sound and smoke? [Namen: Nichts als Schall und Rauch?] *Nervenheilkunde*, *25*, 677-679.

Sowislo, J. F., & Orth, U. (2013). Does low self-esteem predict depression and anxiety? A meta-analysis of longitudinal studies. *Psychological Bulletin*, *139*, 213-240. http://doi.org/10.1037/a0028931

Strack, F., Werth, L., & Deutsch, R. (2006). Reflective and impulsive determinants of consumer behavior. *Journal of Consumer Psychology*, *16*, 205-216. http://doi.org/10.1207/s15327663jcp1603_2

Uhlmann, E. L., Leavitt, K., Menges, J. I., Koopman, J., Howe, M., & Johnson, R. E. (2012). Getting explicit about the implicit: A taxonomy of implicit measures and guide for their use in organizational research. *Organizational Research Methods*, *15*, 553-601. http://doi.org/10.1177/1094428112442750

Valentine, T., Brennen, T., & Bredart, S. (1996). *The cognitive psychology of proper names: On the importance of being Ernest.* London: Routledge.

Van Lancker, D. (1991). Personal relevance and the human right-hemisphere. *Brain and Cognition*, *17*, 64-92. <http://doi.org/10.1016/0278-2626(91)90067-I>

Walther, E., & Langer, T. (2010). Attitude formation and change through association: An evaluative conditioning account. In C. D. William (ed.), *Attitudes and attitude change* (pp. 87-109). New York: Psychology Press.

Wilson, T. D., & Dunn, E. W. (2004). Self-knowledge: Its limits, value, and potential for improvement. *Annual Review of Psychology*, *55*, 493-518. http://doi.org/10.1146/annurev.psych.55.090902.141954

**Irrelevant design**

Abakoumkin, G. (2011). Forming choice preferences the easy way: Order and familiarity effects in elections. *Journal of Applied Social Psychology*, *41*, 2689-2707. http://doi.org/10.1111/j.1559-1816.2011.00845.x

Abel, E. L. (2010). Influence of names on career choices in medicine. *Names-a Journal of Onomastics*, *58*, 65-74. http://doi.org/10.1179/002777310X12682237914945

Abel, E. L., & Kruger, M. L. (2007). Symbolic significance of initials on longevity. *Perceptual and Motor Skills*, *104*, 179-182. http://doi.org/10.2466/PMS-104.1.179-182

Aidman, E. V. (1999). Measuring individual differences in implicit self-concept: initial validation of the self-apperception test. *Personality and Individual Differences*, *27*, 211-228. http://doi.org/10.1016/S0191-8869(98)00234-7

Aidman, E. V., & Carroll, S. M. (2003). Implicit individual differences: Relationships between implicit self-esteem, gender identity, and gender attitudes. *European Journal of Personality*, *17*, 19-37. http://doi.org/10.1002/per.465

Alexopoulos, T., Muller, D., Ric, F., & Marendaz, C. (2012). I, me, mine: Automatic attentional capture by self-related stimuli. *European Journal of Social Psychology*, *42*, 770-779. http://doi.org/10.1002/ejsp.1882

Anseel, F., & Duyck, W. (2009). Implicit letter preferences in job choice: An experimental test of the role of cognitive load. *Journal of Psychology*, *143*, 207-223.

Banse, R. (2001). Affective priming with liked and disliked persons: Prime visibility determines congruency and incongruency effects. *Cognition & Emotion*, *15*, 501-520. http://doi.org/10.1080/02699930126251

Beggan, J. (1992). On the social nature of nonsocial perception: The mere ownership effect. *Journal of Personality and Social Psychology*, *62*, 229-237. http://doi.org/10.1037/0022-3514.62.2.229

Beggan, J. (1994). The preference for gain frames in consumer decision-making. *Journal of Applied Social Psychology*, *24*, 1407-1427. <http://doi.org/10.1111/j.1559-1816.1994.tb01556.x>

Berger, B. A. (1983). *The implementation of an art programme designed to develop cultural awareness among students in an urban native Indian alternate class: A case study*. Unpublished master’s thesis, University of British Columbia, BC.

Bertamini, M., & Lyons, M. (2015). How men and women respond to hypothetical parental discovery: the importance of genetic relatedness. *Evolutionary Psychology*, *13*, 424-434.

Boatright-Horowitz, S. L. (1995). A classroom demonstration of Nuttin’s (1985) ownership effect: The letters of my own first name. *Teaching of Psychology*, *22*, 131-133. <http://doi.org/10.1207/s15328023top2202_12>

Bracey, J. R. (2010). *Socializing race: Parental beliefs and practices in two African American families*. Unpublished doctoral dissertation, University of Illinois at Urbana-Champaign, IL.

Brock, T. (2013). *Autism and transition to work: A thematic analysis of service user experiences*. Unpublished doctoral dissertation, University of Lincoln, UK.

Clark, J. T. (2008). *Developing collaborative leadership: A study of organizational change toward greater collaboration and shared leadership*. Unpublished doctoral dissertation, Antioch University, OH.

De Houwer, J. (2001). A structural and process analysis of the implicit association test. *Journal of Experimental Social Psychology*, *37*, 443-451. <http://doi.org/10.1006/jesp.2000.1464>

DeHart, T., Pelham, B., & Murray, S. (2004). Implicit dependency regulation: Self-esteem, relationship closeness, and implicit evaluations of close others. *Social Cognition*, *22*, 126-146. <http://doi.org/10.1521/soco.22.1.126.30986>

Freeman, E. H. (2012). *Home is where hurt is: Racial socialization, stigma, and well-being in Afro-Brazilian families*. Unpublished doctoral dissertation, Duke University, NC.

Gawronski, B., Bodenhausen, G. V., & Becker, A. P. (2007). I like it, because I like myself: Associative self-anchoring and post-decisional change of implicit evaluations. *Journal of Experimental Social Psychology*, *43*, 221-232. http://doi.org/10.1016/j.jesp.2006.04.001

Griesy, P. H., Cai, H., & Crowson, H. M. (2010). The spectre of Communism in US China policy: Bipartisanship in the American subconscious. *Chinese Journal of International Politics*, *3*, 397-413. <http://doi.org/10.1093/cjip/poq014>

Hasnain, S. K. (2014). *Synchronisation et coordination interpersonelle dans l’interaction Homme-robot*. Unpublished doctoral dissertation, Université d’Evry-Val-d’Essonne, Evry, France.

Howard, D. J., & Kerin, R. A. (2011). The effects of name similarity on message processing and persuasion. *Journal of Experimental Social Psychology*, *47*, 63-71. http://doi.org/10.1016/j.jesp.2010.08.008

Howard, D. J., & Kerin, R. A. (2014). Individual differences in the name similarity effect the role of self-monitoring. *Journal of Individual Differences*, *35*, 111-118. http://doi.org/10.1027/1614-0001/a000133

Huang, Y., Wang, L., & Shi, J. (2009). When do objects become more attractive? The individual and interactive effects of choice and ownership on object evaluation. *Personality and Social Psychology Bulletin*, *35*, 713-722. http://doi.org/10.1177/0146167209333046

Kachersky, L., & Sen, S. (2010). Buyer beware of your shadow: A dual process explanation of name letter branding and avoidance. *NA-Advances in Consumer Research, Volume 37*.

Kitayama, S., & Uchida, Y. (2003). Explicit self-criticism and implicit self-regard: Evaluating self and friend in two cultures. *Journal of Experimental Social Psychology*, *39*, 476-482. http://doi.org/10.1016/S0022-1031(03)00026-X

Klavina, E., Schroeder-Abe, M., & Schuetz, A. (2012). Facets of self-esteem at an implicit level? Investigation of implicit-explicit correlations and development of four IATs. *Personality and Individual Differences*, *53*, 693-698. http://doi.org/10.1016/j.paid.2012.05.028

Knewtson, H. S., & Sias, R. W. (2010). Why Susie owns Starbucks: The name letter effect in security selection. *Journal of Business Research*, *63*, 1324-1327. http://doi.org/10.1016/j.jbusres.2009.12.003

Kocan, S. E., & Curtis, G. J. (2009). Close encounters of the initial kind: Implicit self-esteem, name-letter similarity, and social distance. *Basic and Applied Social Psychology*, *31*, 17-23. http://doi.org/10.1080/01973530802659752

Koole, S. L., Dijksterhuis, A., & van Knippenberg, A. (2001). What’s in a name: Implicit self-esteem and the automatic self. *Journal of Personality and Social Psychology*, *80*, 669-685. http://doi.org/10.1037//0022-3514.80.4.669

Kooti, F., Magno, G., & Weber, I. (2014). The social name-letter effect on online social networks. In L. M. Aiello & D. McFarland (eds.), *Social informatics, Socinfo 2014* (Vol. 8851, pp. 216-227). Barcelona, Spain: Springer.

Lipsitz, A., & Gifford, L. A. (2003). What’s in a name? Better letters if it’s mine! *Teaching of Psychology*, *30*, 58-59.

McDermott, R., & Dozois, D. J. A. (2015). The causal role of attentional bias in a cognitive component of depression. *Journal of Experimental Psychopathology, 6*, 1-13.

Pelham, B., & Mauricio, C. (2015). When Tex and Tess carpenter build houses in Texas: Moderators of implicit egotism. *Self and Identity*, *14*, 692-723. <http://doi.org/10.1080/15298868.2015.1070745>

Rutherford, J. A. (1999). *A formative evaluation of Franklin School*. Unpublished master’s thesis, Virginia Tech, VA.

Schmeichel, B. J., Gailliot, M. T., Filardo, E.-A., McGregor, I., Gitter, S., & Baumeister, R. F. (2009). Terror management theory and self-esteem revisited: The roles of implicit and explicit self-esteem in mortality salience effects. *Journal of Personality and Social Psychology*, *96*, 1077-1087. <http://doi.org/10.1037/a0015091>

Simcock, C. (2016). *Community influences on rural youth wellbeing: Young people’s perspectives*. Unpublished master’s thesis, University of Waikato, New Zealand.

Simonsohn, U. (2011). Spurious? Name similarity effects (implicit egotism) in marriage, job, and moving decisions. *Journal of Personality and Social Psychology*, *101*, 1-24. <http://doi.org/10.1037/a0021990>

Stephens, N. M. (2002). *Can I get a high five and an amen? Successful African Americans testifyin’ to the link between language, literacy, leadership, and their culture*. Unpublished doctoral dissertation, Ohio State University, OH.

Stieger, S., Voracek, M., & Formann, A. K. (2012). How to administer the Initial Preference Task. *European Journal of Personality*, *26*, 63-78. http://doi.org/10.1002/per.823

van den Bergh, O., Vrana, S., & Eelen, P. (1990). Letters from the heart: Affective categorization of letter combinations. *Journal of Experimental Psychology-Learning Memory and Cognition*, *16*, 1153-1161. <http://doi.org/10.1037//0278-7393.16.6.1153>

Wang, J. (2011). *Classical music policy and practice in a British city*. Unpublished doctoral dissertation, University of Nottingham, UK.

Watson, R. J., & Winkelman, J. H. (2005). Short communication: "Perceived ownership” or cognitive dissonance? *European Journal of Social Psychology*, *35*, 403-411. <http://doi.org/10.1002/ejsp.255>

Wentura, D., Kulfanek, M., & Greve, W. (2005). Masked affective priming by name letters: Evidence for a correspondence of explicit and implicit self-esteem. *Journal of Experimental Social Psychology*, *41*, 654-663. http://doi.org/10.1016/j.jesp.2004.02.005

Wheeler, S. C., Petty, R. E., & Bizer, G. Y. (2005). Self-schema matching and attitude change: Situational and dispositional determinants of message elaboration. *Journal of Consumer Research*, *31*, 787-797. http://doi.org/10.1086/426613

Wiersema, D. V., van Harreveld, F., & van der Pligt, J. (2012). Shut your eyes and think of something else: Self-esteem and avoidance when dealing with counter-attitudinal information. *Social Cognition*, *30*, 323-334.

Wu, Y., van Dijk, E., & Zhou, X. (2013). Evaluating self- vs. other-owned objects: The modulatory role of oxytocin. *Biological Psychology*, *92*, 179-184. <http://doi.org/10.1016/j.biopsycho.2012.11.011>

Zeri de Oliveira, C. (2014). *Letramento: Multiplos desfios para a formacao cidada*. Unpublished doctoral dissertation, Universidade de Aveiro, Portugal.

**Irrelevant measure**

Amad, S. (2015). *Self-esteem and aggression: The relationships between explicit-implicit self-esteem, narcissism, and reactive-proactive aggression*. Unpublished doctoral dissertation, Cardiff University, UK.

Anokhina, A. (2015). *Implicit attitudes towards food and the self in sub-clinical eating disorder pathology*. Unpublished doctoral dissertation, University College London, UK.

Ashish, D. (2016). *Self-compassion and the need of self-preservation*. Unpublished doctoral dissertation, University of Arizona, AZ.

Bale, C. (2010). *Attractiveness and self-esteem: A test of sociometer theory*. Unpublished doctoral dissertation, University of Central Lancashire, UK.

Bianchi, I., Savardi, U., & Bertamini, M. (2008). Estimation and representation of head size (people overestimate the size of their head - evidence starting from the 15th century). *British Journal of Psychology*, *99*, 513-531. http://doi.org/10.1348/000712608X304469

Bluemke, M., & Friese, M. (2012). On the validity of idiographic and generic self-concept implicit association tests: A core-concept model. *European Journal of Personality*, *26*, 515-528. <http://doi.org/10.1002/per.850>

Boelens, N. (2014). De samenhang tussen zelfvertrouwen en psychopathie in relatie met etniciteit. Unpublished master’s thesis, Leiden University, Netherlands.

Borton, J. L. S., Crimmins, A. E., Ashby, R. S., & Ruddiman, J. F. (2012). How do individuals with fragile high self-esteem cope with intrusive thoughts following ego threat? *Self and Identity*, *11*, 16-35. <http://doi.org/10.1080/15298868.2010.500935>

Boyce, J. A. (2012). *Experimental exposure to ideal-body media images: Restrained eaters’ self-evaluation, mood and food intake*. Unpublished doctoral dissertation, University of Canterbury, New Zealand.

Brauhardt, A. (2014). *Binge-Eating-Störung: Grundlagen und Psychotherapieprozess*. Unpublished doctoral dissertation, Philipp University of Marburg, Germany.

Brendl, C. M., Chattopadhyay, A., Pelham, B. W., & Carvallo, M. (2005). Name letter branding: Valence transfers when product specific needs are active. *Journal of Consumer Research*, *32*, 405-415. http://doi.org/10.1086/497552

Brinol, P., McCaslin, M. J., & Petty, R. E. (2012). Self-generated persuasion: Effects of the target and direction of arguments. *Journal of Personality and Social Psychology*, *102*, 925-940. <http://doi.org/10.1037/a0027231>

Buechsel, R. K. (2010). *Development of an implicit measure of dispositional forgiveness*. Unpublished doctoral dissertation, Baylor University, TX.

Burke, K. D. (2014). *The effect of social closeness on reactions to social rejection*. Unpublished master’s thesis, Ohio State University, OH.

Canning, A. H. M. (2011). *An investigation of the relationship between self-esteem and aggression in care leavers*. Unpublished doctoral dissertation, Cardiff University, UK.

Carlson, K. A., & Conard, J. M. (2011). The last name effect: How last name influences acquisition timing. *Journal of Consumer Research*, *38*, 300-307. http://doi.org/10.1086/658470

Chen, H., Gallagher, A. C., & Girod, B. (2013). What’s in a name? First names as facial attributes. In *2013 IEEE Conference on Computer Vision and Pattern Recognition* (pp. 3366-3373).

Chen, H., Gallagher, A. C., & Girod, B. (2014). The hidden sides of names-face modeling with first name attributes. *IEEE Transactions on Pattern Analysis and Machine Intelligence*, *36*, 1860-1873. <http://doi.org/10.1109/TPAMI.2014.2302443>

Cialdini, R., & Denicholas, M. (1989). Self-presentation by association. *Journal of Personality and Social Psychology*, *57*, 626-631. <http://doi.org/10.1037//0022-3514.57.4.626>

Conner, T., & Barrett, L. F. (2005). Implicit self-attitudes predict spontaneous affect in daily life. *Emotion*, *5*, 476-488. <http://doi.org/10.1037/1528-3542.5.4.476>

Coulter, K. S., & Grewal, D. (2014). Name-letters and birthday-numbers: Implicit egotism effects in pricing. *Journal of Marketing*, *78*, 102-120. http://doi.org/10.1509/jm.13.0059

Crescentini, C., Urgesi, C., Campanella, F., Eleopra, R., & Fabbro, F. (2014). Effects of an 8-week meditation program on the implicit and explicit attitudes toward religious/spiritual self-representations. *Consciousness and Cognition*, *30*, 266-280. <http://doi.org/10.1016/j.concog.2014.09.013>

Cuellar, R., Jr. (2005). *The validation of the anger implicit association test*. Unpublished doctoral dissertation, Texas A&M University, TX.

Davies, R. (2015). *The conceptualization and assessment of grandiose and vulnerable narcissism: An investigation of common and unique features*. Unpublished doctoral dissertation, University of Melbourne, Australia.

Degner, J., Wentura, D., Gniewosz, B., & Noack, P. (2007). Hostility-related prejudice against Turks in adolescents: Masked affective priming allows for a differentiation of automatic prejudice. *Basic and Applied Social Psychology*, *29*, 245-256.

de Sousa Silva, R. R. (2016). *Dissonância do endogrupo e balanceamento cognitivo – implicações disposicionais e grupais em processos de busca por consistência*. Unpublished master’s thesis, University of Brasilia, Brazil.

Dimaro, L. (2013). *Examining implicit cognition in people with seizures*. Unpublished doctoral dissertation, University of Nottingham, UK.

Du, H. (2012). *Cultural influences on terror management: The roles of self-esteem, norm, and control motivation.* Unpublished master’s thesis, University of Hong Kong, Hong Kong.

Duck, N. J. (2010). *Factors that optimise the credibility of advertisements whilst promoting feelings of emotional well-being and satisfaction*. Unpublished doctoral dissertation, Monash University, Ireland.

Ebert, I. D., Steffens, M. C., von Stuelpnagel, R., & Jelenec, P. (2009). How to like yourself better, or chocolate less: Changing implicit attitudes with one IAT task. *Journal of Experimental Social Psychology*, *45*, 1098-1104. http://doi.org/10.1016/j.jesp.2009.06.008

Eren, A., & Tezel, K. V. (2010). Factors influencing teaching choice, professional plans about teaching, and future time perspective: A mediational analysis. *Teaching and Teacher Education*, *26*, 1416-1428. http://doi.org/10.1016/j.tate.2010.05.001

Farnham, S. D. (1999). *From implicit self-esteem to in-group favoritism*. Unpublished doctoral dissertation, University of Washington, WA.

Farnham, S. D., Greenwald, A. G., & Banaji, M. R. (1999). Implicit self-esteem. In D. Abrams & M. Hogg (eds.), *Social identity and social cognition* (pp. 230-248). Oxford, UK: Blackwell.

Feys, J. (1991). Briefly induced belongingness to self and preference. *European Journal of Social Psychology*, *21*, 547-552. <http://doi.org/10.1002/ejsp.2420210608>

Feys, J. (1995). Mere ownership: Affective self-bias or evaluative conditioning. *European Journal of Social Psychology*, *25*, 559-575. http://doi.org/10.1002/ejsp.2420250507

Franck, E., De Raedt, R., Dereu, M., & Van den Abbeele, D. (2007). Implicit and explicit self-esteem in currently depressed individuals with and without suicidal ideation. *Journal of Behavior Therapy and Experimental Psychiatry*, *38*(1), 75–85. <http://doi.org/10.1016/j.jbtep.2006.05.003>

Frisch, J. U., Haeusser, J. A., van Dick, R., & Mojzisch, A. (2015). The Social Dimension of Stress: Experimental Manipulations of Social Support and Social Identity in the Trier Social Stress Test. *Jove-Journal of Visualized Experiments*, (105), e53101. <http://doi.org/10.3791/53101>

Gillen-O’Neel, C. (2014). *Implicit ethnic group attitude and stereotype development: Causes and consequences*. Unpublished doctoral dissertation, University of California, LA.

Glen, L. S., & Banse, R. (2004). Probing the malleability of implicit and explicit self-esteem: An interview approach. *Cahiers de Psychologie Cognitive-Current Psychology of Cognition*, *22*, 133-158.

Grandfield, T. A. (2007). *Exploring attitudinal and psychophysiological responses towards visible difference: The role of shame, disgust, self-esteem and appearance schema*. Unpublished doctoral dissertation, University of Sheffield, UK.

Halberstadt, J., & Hooton, K. (2008). The affect disruption hypothesis: The effect of analytic thought on the fluency and appeal of art. *Cognition & Emotion*, *22*, 964-976. <http://doi.org/10.1080/02699930701597668>

Hamilton, V. (2012). *An exploration of implicit associations regarding mental illness, self-reported internalised stigma, and their links to help seeking symptom thresholds amongst individuals experiencing depressive symptoms*. Unpublished doctoral dissertation, University of East Anglia, United Arab Emirates.

Hammerl, V. (2011). *Prädiktoren für implizite und explizite rassistische Einstellungen bei Jugendlichen*. Unpublished master’s thesis, University of Vienna, Austria.

Harris, C. J. (2002). *Assessing childrens’ body shape and weight concerns*. Unpublished doctoral dissertation, University of Leeds, UK.

Harte, C. (2015). *Systematic explorations of methodological parameters of the implicit relational assessment procedure (IRAP)*. Unpublished bachelor’s thesis, Maynooth University, Irleand.

Hodgins, H. S., Brown, A. B., & Carver, B. (2007). Autonomy and control motivation and self-esteem. *Self and Identity*, *6*, 189-208. <http://doi.org/10.1080/15298860601118769>

Horn, A. (2009). *The effects of dating site advertisements on the quantity of other partners and the commitment to a love relationship*. Unpublished master’s thesis, University of Utrecht, Netherlands.

Hulme, N. (2010). *Imagery and the self in social phobia*. Unpublished doctoral dissertation, University of Southampton, Southampton, UK.

Justice, L. M., Pence, K., Bowles, R. B., & Wiggins, A. (2006). An investigation of four hypotheses concerning the order by which 4-year-old children learn the alphabet letters. *Early Childhood Research Quarterly*, *21*, 374-389. http://doi.org/10.1016/j.ecresq.2006.07.010

Kachersky, L., & Carnevale, M. (2015). Effects of pronoun brand name perspective and positioning on brand attitude. *Journal of Product and Brand Management*, *24*, 157-164. http://doi.org/10.1108/JPBM-02-2014-0495

Kalyanaraman, S., & Sundar, S. S. (2006). The psychological appeal of personalized content in web portals: Does customization affect attitudes and behavior? *Journal of Communication*, *56*, 110-132. <http://doi.org/10.1111/j.1460-2466.2006.00006.x>

Kostiuk, N. E. (2012). *Implicit and explicit self-esteem, narcissism, risk, and psychopathy in a forensic population*. Unpublished doctoral dissertation, University of Alberta, AB.

Krueger, J. (1998). Enhancement bias in descriptions of self and others. *Personality and Social Psychology Bulletin*, *24*, 505-516. http://doi.org/10.1177/0146167298245006

Kulig, J. W. (2013). What’s in a name? Our false uniqueness! *British Journal of Social Psychology*, *52*, 173-179. <http://doi.org/10.1111/bjso.12001>

Lam, C. P. (2016). *Dialectical relationship thinking: Examination of partner evaluation and partner knowledge organization across cultures*. Unpublished doctoral dissertation, Iowa State University, IA.

Law, H. (2014). *Understanding recovery in psychosis*. Unpublished doctoral dissertation, University of Manchester, UK.

Lechner, K. (2013). *When time becomes distorted: A narcissist’s view*. Unpublished master’s thesis, Eastern Washington University, WA.

Lemmens, L. H. J. M., Roefs, A., Arntz, A., van Teeseling, H. C., Peeters, F., & Huibers, M. J. H. (2014). The value of an implicit self-associative measure specific to core beliefs of depression. *Journal of Behavior Therapy and Experimental Psychiatry*, *45*, 196-202. <http://doi.org/10.1016/j.jbtep.2013.10.006>

Leviston, Z. (2013). The social and psychological functions of responses to climate change. Unpublished doctoral dissertation, Curtin University, Australia.

Lippman, A. D. P. (2012). Homeless young adults: An exploratory study examining resiliency and coping. Unpublished doctoral dissertation, University of Texas at Austin, TX.

Lockwood, P., Burton, C., & Boersma, K. (2011). Tampering with tradition: Rationales concerning women’s married names and children’s surnames. *Sex Roles*, *65*, 827-839. <http://doi.org/10.1007/s11199-011-0034-1>

Maniaci, M. R. (2015). *For better, for worse: Discrepancies between implicit and explicit evaluations in newlywed marriage.* Unpublished doctoral dissertation, University of Rochester, NY.

McKenna, M. (2013). Implicit theories of intelligence: Effects on attributions, grade average, task choice and test scores. Unpublished doctoral dissertation, University of Edinburgh, UK.

Meites, T. M. (2009). *Implicit self-esteem in adolescents with parental history of depression*. Unpublished master’s thesis, University of Kansas, KS.

Miller, K. (2015). *An examination of whether hearing a display of self-compassion in someone else impacts one’s own level of self-compassion*. Unpublished master’s thesis, Waterloo University, ON.

Miyamoto, R., & Kikuchi, Y. (2012). Gender differences of brain activity in the conflicts based on implicit self-esteem. *PLOS ONE*, *7*, e37901. <http://doi.org/10.1371/journal.pone.0037901>

Mogilner, C., & Aaker, J. (2009). “The Time vs. Money Effect”: Shifting product attitudes and decisions through personal connection. *Journal of Consumer Research*, *36*, 277-291. <http://doi.org/10.1086/597161>

Nash, A. S. (2005). *Understanding children’s thinking about alcohol advertisements on television: A cognitive developmental approach*. Unpublished doctoral dissertation, University of Hertfordshire, UK.

Nevicky, B. (2012). *Narcissistic leaders: The appearance of success*. Unpublished doctoral dissertation, University of Amsterdam, Netherlands.

Newman, L. S., Hernandez, W., Bakina, D. A., & Rutchick, A. M. (2009). Implicit egotism on the baseball diamond: Why Peter Piper prefers to pitch for the Pittsburgh Pirates. *Names-a Journal of Onomastics*, *57*, 175-179. <http://doi.org/10.1179/175622709X462478>

Oakes, M. A., Brown, J. D., & Cai, H. (2008). Implicit and explicit self-esteem: Measure for measure. *Social Cognition*, *26*, 778-790.

Ohnesorge, C., & Van Lancker, D. (2001). Cerebral laterality for famous proper nouns: Visual recognition by normal subjects. *Brain and Language*, *77*, 135-165. <http://doi.org/10.1006/brln.2000.2365>

Olyedemi, M. (2013). *Towards a psychology of mixed-race identity development in the United Kingdom*. Unpublished doctoral dissertation, Brunel University, UK.

Pannese, A., & Hirsch, J. (2013). Unconscious neural specificity for “self” and the brainstem. *Journal of Consciousness Studies*, *20*, 169-179.

Pavlickova, H. (2013). *Psychological vulnerability in bipolar disorder*. Unpublished doctoral thesis, Bangor University, UK.

Pearce, E. (2002). *Attributional style and self-concept in sex offenders with persecutory delusions: An exploratory study*. Unpublished doctoral dissertation, University of Wales, UK.

Peham, J. A. (2015). Die Verhaltensvorhersage impliziter Selbstwertmaße am Beispiel des IAT und CAT. *Unpublished master’s thesis, University of Vienna, Austria.*

Pepper, R. (2013). *Using the implicit association test to assess attachment, self-esteem, and implicit theories among sexual offenders*. Unpublished doctoral dissertation, University of Cardiff, UK.

Perkins, A. W., & Forehand, M. R. (2012). Implicit self-referencing: The effect of nonvolitional self-association on brand and product attitude. *Journal of Consumer Research*, *39*, 142-156. <http://doi.org/10.1086/662069>

Peterse, T. (2014). *Psychopathische kenmerken bij adolescenten en de relatie met zelfwaardering het modererende effect van etnische afkomst op deze relatie*. Unpublished master’s thesis, Leiden University, Netherlands.

Pham, D. (2008). *Narcissism och syskonplacering hos gymnasieelever.* Unpublished bachelor’s thesis, Linneuniversitetet, Sweden.

Polman, E., Pollmann, M. M. H., & Poehlman, T. A. (2013). The name-letter-effect in groups: sharing initials with group members increases the quality of group work. *PLOS ONE*, *8*, e79039. <http://doi.org/10.1371/journal.pone.0079039>

Poteau, S. R. (2009). *Implicit cognition and terror management theory: The utility of indirect measurement in understanding death-related defense mechanisms*. Unpublished doctoral dissertation, Temple University, Philadelphia, PA.

Pruneddu, A. (2013). *Implicit theories and Q-sort: Personality change in emerging adulthood*. Unpublished doctoral thesis, University of York, UK.

Quirin, M., Kazen, M., & Kuhl, J. (2009). When nonsense sounds happy or helpless: The implicit positive and negative affect test (IPANAT). *Journal of Personality and Social Psychology*, *97*, 500-516. <http://doi.org/10.1037/a0016063>

Rai, L. (2015). *The measurement of implicit responses to life and death: Implications for sub-clinical psychopathology.* Unpublished master’s thesis, Maynooth University, Ireland*.*

Ravary, A. (2016). *Insecurity about aging and attentional biases towards social rejection*. Unpublished master’s thesis, McGill University, QC.

Reis-Bergan, M. J. (2000). *Blame for past behavior versus threat to future freedom: Reaction to anti-binge drinking messages*. Unpublished doctoral dissertation, Iowa State University, IA.

Riketta, M., & Dauenheimer, D. (2003). Manipulating self-esteem with subliminally presented words. *European Journal of Social Psychology*, *33*, 679-699. <http://doi.org/10.1002/ejsp.179>

Ritter, K. (2013). *The narcissistic personality disorder: Empirical studies*. Unpublished doctoral dissertation, Humboldt University Berlin, Germany.

Ritter, K., Roepke, S., Merkl, A., Heuser, I., Fydrich, T., & Lammers, C.-H. (2010). Komorbiditäten bei Patienten mit einer Narzisstischen Persönlichkeitsstörung im Vergleich zu Patienten mit einer Borderline-Persönlichkeitsstörung [Comorbidity in patients with narcissistic personality disorder in comparison to patients with borderline personality disorder]. *Journal of Behavior Therapy and Experimental Psychiatry, 44*, 37-47.

Ritter, K., Vater, A., Rüsch, N., Schröder-Abé, M., Schütz, A., Fydrich, T., Lammers, C.-M., & Roepke, S. (2014). Shame in patients with narcissistic personality disorder. *Psychiatry Research, 215*, 429-437.

Rousseau, J. (2009). *Relations interethniques et identite a l’ecole Primaire: Effet du groupe ethnique, de l’âge et du contexte scolaire*. Unpublished doctoral dissertation, Université de Bordeaux, France.

Rudolph, A., Schröder-Abé, M., Riketta, M., & Schütz, A. (2009). Easier when done than said! Implicit self-esteem predicts observed or spontaneous behavior, but not self-reported or controlled behavior. *Zeitschrift für Psychologie / Journal of Psychology, 218*, 12-19.

Russ, S. L. (2009). *Identity relativity: Linking stereotype threat and social comparison as parallel processes*. Unpublished doctoral dissertation, University of Minnesota, MN.

Ryan, J. P. (2009). *Self-esteem, failure feedback, and physiological reactivity: Implications for working memory and aggression.* Unpublished doctoral dissertation, Georgia State University, GA.

Sargeant, C. C. (2013). *Examining the relationship between sources of self-concept and forms of aggression in adolescence.* Unpublished doctoral dissertation, University of Southampton, Southampton, UK.

Schroeder-Abe, M., Rudolph, A., & Schuetz, A. (2007). High implicit self-esteem is not necessarily advantageous: Discrepancies between explicit and implicit self-esteem and their relationship with anger expression and psychological health. *European Journal of Personality*, *21*, 319-339. http://doi.org/10.1002/per.626

Sears, D., & Funk, C. (1991). The role of self-interest in social and political-attitudes. *Advances in Experimental Social Psychology*, *24*, 1-91. <http://doi.org/10.1016/S0065-2601(08)60327-5>

Simon, E. (2011). *Zur Praktikabilität des IAT in der Fallbehandlung: Machbarkeit, Akzeptanz und wiederholte Retest-Reliabilität im Vergleich zu ILICA*. Unpublished master’s thesis, University of Vienna, Austria.

Skinner, S. (2011). *You hurt me, I’ll hurt you: The prediction of aggression based on the interaction between an ego threat, fragile high self-esteem, and narcissistic traits*. Unpublished master’s thesis, University of North Carolina, Chapel Hill, NC.

Skorek, M. (2011). Effects of exposure to idealized body portrayals in an ethnically diverse sample of men and women. Unpublished doctoral dissertation, University of California, CA.

Smurda, J. D. (2001). Effects of threat to a valued social identity on implicit self-esteem and discrimination. Unpublished master’s thesis, California State University, CA.

Smurda, J. D., Wittig, M. A., & Gokalp, G. (2006). Effects of threat to a valued social identity on implicit self-esteem and discrimination. Group Processes & Intergroup Relations, 9, 181-197.

Spardello, M. (2012). *Creativity beliefs of elementary students: Self-efficacy, self-esteem and beliefs in between*. Unpublished master’s thesis, Georgia State University, GA.

Storek, J. (2011). The hubris and humility effect and the domain-masculine intelligence type: Exploration of determinants of gender differences in self-estimation of ability. Unpublished doctoral dissertation, University College London, UK.

Sueyoshi, M. (1996). *Delusions, cognitive biases and emotional priming*. Unpublished master’s thesis, Durham University, UK.

Robinson, L. J. (2010). *Neuropsychological performance, emotion processing and psychosocial function in bipolar disorder*. Unpublished doctoral dissertation, Newcastle University, UK.

Spalding, L. R., & Hardin, C. D. (1999). Unconscious unease and self-handicapping: Behavioral consequences of individual differences in implicit and explicit self-esteem. *Psychological Science*, *10*, 535-539. http://doi.org/10.1111/1467-9280.00202

Stieger, S., Burger, C., Schiller, F. R., Schulze, E. K., & Voracek, M. (2014). Measuring implicit gender-role orientation: the gender initial preference task. *Journal of Personality Assessment*, *96*, 358-367. <http://doi.org/10.1080/00223891.2013.825622>

Stieger, S., & Krizan, Z. (2013). Cultural influences on number preferences: Christmas and grading systems. *Psychological Record*, *63*, 185-191.

Stieger, S., & Voracek, M. (2014). Not only dogs resemble their owners, cars do, too. *Swiss Journal of Psychology*, *73*, 111-117. http://doi.org/10.1024/1421-0185/a000130

Szeto, A. C. H., Sorrentino, R. M., Yasunaga, S., Otsubo, Y., Kouhara, S., & Sasayama, I. (2009). Using the implicit association test across cultures: A case of implicit self-esteem in Japan and Canada. *Asian Journal of Social Psychology*, *12*, 211-220. <http://doi.org/10.1111/j.1467-839X.2009.01286.x>

Talati, Z. (2013). *Death as the ultimate loss of control: Comparing the effects of mortality salience and loss of control through an examination of moderators and outcomes*. Unpublished doctoral dissertations, University of Western Australia, Australia.

Teige-Mocigemba, S. (2008). *Beyond structural problems of the Implicit Association Test (IAT): Approaches to reduce contaminations of IAT effects*. Unpublished doctoral dissertation, Albert-Ludwigs University Freiburg, Germany.

Thomas, B. (2015). *Dark side personality IATS: Development of non-bipolar valence-balanced implicit measures.* Unpublished master’s thesis, Missouri State University, MO.

Thompson-Leonardelli, K. I. (2002). *Is thin in: The influence of sociocultural variables on the development of body image and disordered eating in African and European American college women*. Unpublished doctoral dissertation, Ohio State University, OH.

Topolinski, S., & Boecker, L. (2016). Minimal conditions of motor inductions of approach-avoidance states: The case of oral movements. *Journal of experimental Psychology: General, 145*, 1589-1603.

Tran, T. B. (2012). *Rumination and emotional adjustment: The role of social networking sites*. Unpublished doctoral dissertation, University of Miami, FL.

Treiman, R., & Broderick, V. (1998). What’s in a name: Children’s knowledge about the letters in their own names. *Journal of Experimental Child Psychology*, *70*, 97-116. http://doi.org/10.1006/jecp.1998.2448

Treiman, R., Schmidt, J., Decker, K., Robins, S., Levine, S. C., & Demir, O. E. (2015). Parents’ talk about letters with their young children. *Child Development*, *86*, 1406-1418. http://doi.org/10.1111/cdev.12385

Turk, D. J., Gillespie-Smith, K., Krigolson, O. E., Havard, C., Conway, M. A., & Cunningham, S. J. (2015). Selfish learning: The impact of self-referential encoding on children’s literacy attainment. *Learning and Instruction*, *40*, 54–60. <http://doi.org/10.1016/j.learninstruc.2015.08.001>

Turnbull, K. L. P., Bowles, R. P., Skibbe, L. E., Justice, L. M., & Wiggins, A. K. (2010). Theoretical Explanations for Preschoolers’ Lowercase Alphabet Knowledge. *Journal of Speech Language and Hearing Research*, *53*(6), 1757–1768. <http://doi.org/10.1044/1092-4388(2010/09-0093)>

Turner, M. (2012). *Self-focused attention and appearance-related comparisons in body dysmorphic disorder*. Unpublished doctoral dissertation, Kings College London, UK.

Uchida, Y., & Kitayama, S. (2000). Implicit self-attachment in Japan: an examination with an implicit association test. In G. Hatano, N. Okada, & H. Tanabe (eds.). *Proceedings of the 13th Toyota Conference on Affective Minds*. Oxford: Elsevier Science.

Uhlmann, E. L., & Nosek, B. A. (2012). My culture made me do it lay theories of responsibility for automatic prejudice. *Social Psychology*, *43*, 108-113. <http://doi.org/10.1027/1864-9335/a000089>

van Dijk, S. (2013). *De ontwikkeling van secundaire psychopathische kenmerken bij Marokkaanse jongeren*. Unpublished master’s thesis, Leiden University, Netherlands.

van Midden, H. (2014). *Narcistische eigenschappen en een laag zelfbeeld in relatie tot gedragsproblemen bij jongeren van 12-17 jaar*. Unpublished master’s thesis, Leiden University, Netherlands.

van Tilburg, W. A. P., & Igou, E. R. (2014). The impact of middle names: Middle name initials enhance evaluations of intellectual performance. *European Journal of Social Psychology*, *44*, 400-411. <http://doi.org/10.1002/ejsp.2026>

van Wijk, I. A. M. (2014). *The behavioral and physiological effect(s) of social defeat among male adolescents: A pilot study*. Unpublished master’s thesis, Leiden University, Netherlands.

Vater, A. (2012). *Reflections in a cloudy pond: Definition and measurement of narcissistic personality disorder*. Unpublished doctoral dissertation, Free University Berlin, Germany.

Vater, A., Ritter, K., Schroeder-Abe, M., Schuetz, A., Lammers, C.-H., Bosson, J. K., & Roepke, S. (2013). When grandiosity and vulnerability collide: Implicit and explicit self-esteem in patients with narcissistic personality disorder. *Journal of Behavior Therapy and Experimental Psychiatry*, *44*, 37-47. <http://doi.org/10.1016/j.jbtep.2012.07.001>

Vater, A., Schröder-Abé, M., Ritter, K., Schulze, L., Renneberg, B, Bosson, J., Röpke, S. (2012). The Narcissistic Personality Inventory: A useful tool for assessing pathological narcissism? Evidence from patients with Narcissistic Personality Disorder. Journal for Personality Assessment. *Journal of Personality Assessment, 93*, 301-308.

Veletanlic, M. (2007)*. Assessing attachment models using the implicit association test*. Unpublished doctoral dissertation, Kristianstad University, Sweden.

Walther, E. (2002). Guilty by mere association: Evaluative conditioning and the spreading attitude effect. *Journal of Personality and Social Psychology*, *82*, 919-934. <http://doi.org/10.1037//0022-3514.82.6.919>

Walther, E., & Trasselli, C. (2003). I like her, because I like myself: Self-evaluation as a source of interpersonal attitudes. *Experimental Psychology*, *50*, 239-246. <http://doi.org/10.1027//1618-3169.50.4.239>

Whitfield, M. L. (2006). *Intuition and correspondence between implicit and explicit self-esteem*. Unpublished master’s thesis, Wilfrid Laurier University, ON.

Windsor-Shellard, B. (2015). *On feeling torn about one’s sexuality: The effect of explicit-implicit sexual orientation ambivalence*. Unpublished doctoral dissertation, Cardiff University, UK.

**No explicit self-esteem measure**

Bone, S. A., Christensen, G. L., & Williams, J. D. (2014). Rejected, shackled, and alone: Rhe impact of systemic restricted choice on minority consumers’ construction of self. *Journal of Consumer Research*, *41*, 451-474. http://doi.org/10.1086/676689

Boyd, R. L., & Robinson, M. D. (2015). Not sugar and spice and everything nice: name-letter preferences as a predictor of daily hostile behavior. *Self and Identity*, *14*, 147-156. http://doi.org/10.1080/15298868.2014.965731

Brownlow, S., Attea, M. F., Makransky, J. A., & Lopez, A. O. (2007). Name letter matching and implicit egotism: Friends as self-extensions. *Social Behavior and Personality*, *35*, 525-535. http://doi.org/10.2224/sbp.2007.35.4.525

Chen, S., & Boucher, H. C. (2008). Relational selves as self-affirmational resources. *Journal of Research in Personality*, *42*, 716-733. <http://doi.org/10.1016/j.jrp.2007.09.006>

Ciani, K. D., & Sheldon, K. M. (2010). A versus F: The effects of implicit letter priming on cognitive performance. *British Journal of Educational Psychology, 80*, 99-119.

De Raedt, R., Schacht, R., Franck, E., & De Houwer, J. (2006). Self-esteem and depression revisited: Implicit positive self-esteem in depressed patients? *Behaviour Research and Therapy*, *44*, 1017-1028. http://doi.org/10.1016/j.brat.2005.08.003

Dijksterhuis, A. (2004). I like myself but I don’t know why: Enhancing implicit self-esteem by subliminal evaluative conditioning*. Journal of Personality and Social Psychology, 86,* 345-355.

Holland, R. W., Wennekers, A. M., Bijlstra, G., Jongenelen, M. M., & van Knippenberg, A. (2009). Self-symbols as implicit motivators. *Social Cognition*, *27*, 579-600.

Hoorens, V., & Nuttin, J. (1993). Overvaluation of own attributes: Mere ownership or subjective frequency. *Social Cognition*, *11*, 177-200.

Hoorens, V., Nuttin, J., Herman, I., & Pavakanun, U. (1990). Mastery pleasure versus mere ownership: A quasi-experimental cross-cultural and cross-alphabetical test of the name letter effect. *European Journal of Social Psychology*, *20*, 181-205.

Hoorens, V., Remmers, N., & van de Riet, K. (1999). Time is an amazingly variable amount of money: Endowment and ownership effects in the subjective value of working time. *Journal of Economic Psychology*, *20*, 383-405. <http://doi.org/10.1016/S0167-4870(99)00014-8>

Hoorens, V., & Todorova, E. (1988). The name letter effect: Attachment to self or primacy of own name writing. *European Journal of Social Psychology*, *18*, 365-368. <http://doi.org/10.1002/ejsp.2420180406>

Jraidi, I., & Frasson, C. (2010). Subliminally enhancing self-esteem: Impact on learner performance and affective state. In: Aleven V., Kay J., & Mostow J. (eds.), *Intelligent Tutoring Systems*. ITS 2010: Lecture Notes in Computer Science (Vol. 6095, pp. 11-20). Berlin, Germany: Springer.

Kitayama, S., & Karasawa, M. (1997). Implicit self-esteem in Japan: Name letters and birthday numbers. *Personality and Social Psychology Bulletin*, *23*, 736-742. http://doi.org/10.1177/0146167297237006

Koole, S. L., Smeets, K., van Knippenberg, A., & Dijksterhuis, A. (1999). The cessation of rumination through self-affirmation. *Journal of Personality and Social Psychology*, *77*, 111-125. <http://doi.org/10.1037/0022-3514.77.1.111>

LeBel, E. P., & Campbell, L. (2009). Implicit partner affect, relationship satisfaction, and the prediction of romantic breakup. *Journal of Experimental Social Psychology*, *45*, 1291-1294. http://doi.org/10.1016/j.jesp.2009.07.003

LeBel, E. P., & Campbell, L. (2013). The interactive role of implicit and explicit partner evaluations on ongoing affective and behavioral romantic realities. *Social Psychological and Personality Science*, *4*, 167-174. <http://doi.org/10.1177/1948550612448196>

Mackinnon, S. (2009). *Birds of a feather sit together: Physical similarity predicts seating choice*. Unpublished master’s thesis, Wilfrid Laurier University, Waterloo, ON.

Nuttin, J. (1987). Affective consequences of mere ownership: The name letter effect in 12 European languages. *European Journal of Social Psychology*, *17*(4), 381–402. <http://doi.org/10.1002/ejsp.2420170402>

Peterson, J. L., Bellows, A., & Peterson, S. (2015). Promoting connection: Perspective-taking improves relationship closeness and perceived regard in participants with low implicit self-esteem. *Journal of Experimental Social Psychology*, *56*, 160-164. <http://doi.org/10.1016/j.jesp.2014.09.013>

Phelan, J. E., & Rudman, L. A. (2010). Reactions to ethnic deviance: The role of backlash in racial stereotype maintenance. *Journal of Personality and Social Psychology*, *99*, 265-281. <http://doi.org/10.1037/a0018304>

Prewitt-Freilino, J. L., & Bosson, J. K. (2008). Defending the self against identity misclassification. *Self and Identity*, *7*, 168-183. <http://doi.org/10.1080/17405620701330706>

Sakellaropoulo, M. (2015). *The hidden sides of self-esteem: Two dimensions of implicit self-esteem and their relation to narcissistic reactions*. Unpublished master’s thesis, Mc Gill University, Montreal, QC.

Sakellaropoulo, M., & Baldwin, M. W. (2007). The hidden sides of self-esteem: Two dimensions of implicit self-esteem and their relation to narcissistic reactions. *Journal of Experimental Social Psychology*, *43*, 995-1001. http://doi.org/10.1016/j.jesp.2006.10.009

Stieger, S. (2010). Name-letter branding under scrutiny: Real products, new algorithms, and the probability of buying. *Perceptual and Motor Skills*, *110*, 1089-1097. <http://doi.org/10.2466/01.07.PMS.110.C.1089-1097>

Stieger, S., & LeBel, E. P. (2012). Name-letter preferences for new last name and abandoned birth name initials in the context of name-change via marriage. *Social Psychology*, *43*, 7-13. <http://doi.org/10.1027/1864-9335/a000075>

Verplanken, B., Friborg, O., Wang, C. E., Trafimow, D., & Woolf, K. (2007). Mental habits: Metacognitive reflection on negative self-thinking. *Journal of Personality and Social Psychology*, *92*, 526-541. <http://doi.org/10.1037/0022-3514.92.3.526>

Yamaguchi, M. (2011). *Pitfalls of extrinsic goal pursuit: Wellbeing, life goals, and self-focused attention*. Unpublished doctoral dissertation, University of Otago, New Zealand.

**Insufficient reporting of parameters**

Baccus, J. R. (2005). *Testing a cognitive model of implicit self-esteem through evaluative conditioning*. Unpublished doctoral dissertation, McGill University, QC.

Dehart, T., Pelham, B., Fiedorowicz, L., Carvallo, M., & Gabriel, S. (2011). Including others in the implicit self: Implicit evaluation of significant others. *Self and Identity*, *10*, 127-135. <http://doi.org/10.1080/15298861003687880>

Eaton, J., Struthers, C. W., Shomrony, A., & Santelli, A. G. (2007). When apologies fail: The moderating effects of implicit and explicit self-esteem on apology and forgiveness. *Self and Identity, 6*, 209-222.

Epley, N., & Whitchurch, E. (2008). Mirror, mirror on the wall: Enhancement in self-recognition. *Personality and Social Psychology Bulletin*, *34*, 1159-1170. <http://doi.org/10.1177/0146167208318601>

Govorun, O. (2006). *The effect of meditation on the relation on the relation between implicit and explicit self-esteem*. Unpublished doctoral dissertation, Ohio State University, OH.

Grumm, M., Nestler, S., & von Collani, G. (2009). Changing explicit and implicit attitudes: The case of self-esteem. *Journal of Experimental Social Psychology*, *45*, 327-335. <http://doi.org/10.1016/j.jesp.2008.10.006>

Gurari, I., Strube, M. J., & Hetts, J. J. (2009). Death? Be proud! The ironic effects of terror salience on implicit self-esteem. *Journal of Applied Social Psychology*, *39*, 494-507. <http://doi.org/10.1111/j.1559-1816.2008.00448.x>

Pavlova, B., Uher, R., Dennington, L., Wright, K., & Donaldson, C. (2011). Reactivity of affect and self-esteem during remission in bipolar affective disorder: An experimental investigation. *Journal of Affective Disorders*, *134*, 102-111. <http://doi.org/10.1016/j.jad.2011.04.023>

Pena, R. J. (2013). *Secure and insecure high self-esteem and social identity affirmation in response to belongingness threats*. Unpublished master’s thesis, Loyola University Chicago, IL.

Reyes, N. (2012). *Perceived partner commitment and implicit self-esteem predicts connectedness accessibility in response to relationship threat*. Unpublished master’s thesis, Loyola University Chicago, IL.

Smeijers, D., Vrijsen, J. N., van Oostrom, I., Isaac, L., Speckens, A., Becker, E. S., & Rinck, M. (2017). Implicit and explicit self-esteem in remitted depressed patients. *Journal of Behavior Therapy and Experimental Psychiatry, 54*, 301-306.

Svaldi, J., Zimmermann, S., & Naumann, E. (2012). The impact of an implicit manipulation of self-esteem on body dissatisfaction. *Journal of Behavior Therapy and Experimental Psychiatry*, *43*, 581-586. <http://doi.org/10.1016/j.jbtep.2011.08.003>

Todd, N. L. (2012). *Is self-esteem embodied? The influence of vertical orientation on self-esteem and domain specific sociometers*. Unpublished master’s thesis, University of Otago, New Zealand.

Verkuyten, M. (2005). The puzzle of high self-esteem among ethnic minorities: Comparing explicit and implicit self-esteem. *Self & Identity, 4*, 177-192.

**Irrelevant sample (i.e., patients)**

Franck, E., De Raedt, R., & De Houwer, J. (2007). Implicit but not explicit self-esteem predicts future depressive symptomatology. *Behaviour Research and Therapy*, *45*, 2448-2455. http://doi.org/10.1016/j.brat.2007.01.008

Vater, A., Schroeder-Abe, M., Schuetz, A., Lammers, C.-H., & Roepke, S. (2010). Discrepancies between explicit and implicit self-esteem are linked to symptom severity in borderline personality disorder. *Journal of Behavior Therapy and Experimental Psychiatry*, *41*, 357-364. http://doi.org/10.1016/j.jbtep.2010.03.007

Wegener, I., Geiser, F., Alfter, S., Mierke, J., Imbierowicz, K., Kleiman, A., … Conrad, R. (2015). Changes of explicitly and implicitly measured self-esteem in the treatment of major depression: Evidence for implicit self-esteem compensation. *Comprehensive Psychiatry*, *58*, 57-67. http://doi.org/10.1016/j.comppsych.2014.12.001

**Irrelevant age (mean age < 18 years)**

Corenblum, B. (2014a). Development of racial-ethnic identity among first nation children. *Journal of Youth and Adolescence*, *43*, 356-374. http://doi.org/10.1007/s10964-013-0007-5

Corenblum, B. (2014b). Relationships between racial-ethnic identity, self-esteem and in-group attitudes among first nation children. *Journal of Youth and Adolescence*, *43*, 387-404. http://doi.org/10.1007/s10964-013-0081-8

Corenblum, B., & Armstrong, H. D. (2012). Racial-ethnic identity development in children in a racial-ethnic minority group. *Canadian Journal of Behavioural Science-Revue Canadienne des Sciences du Comportement*, *44*, 124-137. <http://doi.org/10.1037/a0027154>

Huijding, J., Bos, A. E. R., & Muris, P. (2011). Enhancing implicit self-esteem in children: Can a smile make you feel worthwhile? *Netherlands Journal of Psychology, 66*, 11-25.

Mamic, T. (2005). *Svälvet under adolescensen*. Unpublished master’s thesis, Kristianstad University College, Sweden.

**Dependent data**

Albers, L. W. A. (2010). *Double you? Function and form of implicit and explicit self-esteem*. Unpublished doctoral dissertation, University of Amsterdam, Netherlands.

Falk, C. F., Heine, S. J., Takemura, K., Zhang, C. X. J., & Hsu, C.-W. (2014). Are implicit self-esteem measures valid for assessing individual and cultural differences? *Journal of Personality, 83*, 56-68.

Frick, S. (2013). *Impliziter Selbstwert und Affekt über die Lebensspanne.* Unpublished master’s thesis, University of Vienna, Austria.

Hoorens, V. (2014). The validity of name-letter preferences (Unpublished data). (personal communication: V. Hoorens to J.P., September 9, 2016)

Phillips, W. J., & Hine, D. W. (2013). Exploring the factor structure of implicit and explicit cognitions associated with depression. *Assessment*, *20*, 474-483. http://doi.org/10.1177/1073191112437595

Phillips, W. J., & Hine, D. W. (2016). En route to depression: Self-esteem discrepancies and habitual rumination. *Journal of Personality*, *84*, 79-90. <http://doi.org/10.1111/jopy.12141>

Rudolph, A. (2009). *Measures of implicit self-esteem: Psychometric properties and the prediction of anxious, self-confident and defensive behavior*. Unpublished doctoral dissertation, Technical University of Chemnitz, Germany.

Schröder-Abé, M. (2007). *Discrepancies between implicit and explicit self-esteem: Measurement issues and relations to health and defensiveness*. Unpublished doctoral dissertation, Technical University of Chemnitz, Germany.

Sedikides, C. (2007). *The fragility versus robustness of narcissistic identity*. Research Report, Swindon: ESRC.

**Excluded due to language**

Fujii, T. (2014). Relationships between explicit/implicit self-esteem discrepancy and measures of depression, loneliness, and in-group favoritism. *Shinrigaku Kenkyu, 85*, 93-99.

Gu, N., Yang, H., & Wang, F. (2014). Name letter effect: An index of implicit self-esteem. *Advances in Psychology, 4*, 284-293.

Sparen, J., & Ryefalk, R. (2013). *Äh! Vadå nervös? Kör nu bara! En studie om hur lärare och elever kan arbeta i gymnasieskolan för att lära sig hantera rampfeber*. Unpublished master’s thesis, Örebro University, Sweden.

Sverrisdottir, I. (2010). *Að finna sjálfið, eða finna það upp Viðmið um sjálfið í sálfræði og heimspeki*. Unpublished bachelor’s thesis, University of Iceland, Iceland.

Vianey, T.-K. L. (2013). *Intuicao e a correspondencia entre autoestima implicita e explicita.* Unpublished master’s thesis, University of Brasilia, Brasilia, Brazil.

Zhen, Z. (2003). Oversea researches on implicit self-esteem. *Advances in Psychological Science*, *11*, 551-554.

**Retracted paper**

Noordewier, M. K., van Horen, F., Ruys, K. I., & Stapel, D. A. (2010). What’s in a name? 361.708 euros: The effects of marital name change (Retracted article. See vol. 34, pg. 192, 2012). *Basic and Applied Social Psychology*, *32*, 17-25. http://doi.org/10.1080/01973530903539812
