# Supplementary material for: Indirect (implicit) and direct (explicit) self-esteem measures are virtually unrelated: A meta-analysis of the initial preference task
Source: PLoS One. 2018 Sep 6;13(9):e0202873. doi: 10.1371/journal.pone.0202873 (PMC6126831; doi:10.1371/journal.pone.0202873)
Supplement: S2 Text — (DOCX) [file pone.0202873.s002.docx]

library(metafor)

library(foreign)

library(car)

library(pbkrtest)

library(faraway)

##Specify data as "c"

## Variable names as available from Supplementary Table 2

## r = zero order correlations between expl. and impl. measures

## sample_size = n within each sample

## publication_status = published vs. unpublished studies

## administration = pen and paper vs. computer

## alg_comp = b-algorithm vs. other algorithm

## bnt = yes vs. no ## (no indicates no inclusion of the birthday preference into the calculation of the IPT)

## likingvsbeauty = liking vs. beauty ## (rating type)

## singlevsdouble = single vs. double

## order = 1 vs. 2 ## expl-impl (order 1) vs. impl-expl (order 2)

## publication_year = year of publication

## male_ratio = % men

## publication_status = published vs. unpublished ## (publication status)

##random effects model overall

data<-escalc(ri=r,ni=sample_size,data=c,measure="ZCOR")

res<-rma(yi,vi,data=data)

res.predict<-predict(res,transf=transf.ztor)

res.predict

res

##zs need to be transformed to rs for interpretation using the predict function (exp(2*z)-1)/(exp(2*z)+1)

##Sensitivity analyses

l1<-leave1out(res)

summary(l1$estimate)

##random effects model for only published samples

res1<-rma(yi,vi,data=data,subset=publication_status=="published")

res1

predict(res1,transf=transf.ztor)

##Sensitivity analyses

l2<-leave1out(res1)

summary(l2$estimate)

##random effects model for only unpublished samples

res2<-rma(yi,vi,data=data,subset=publication_status=="unpublished")

res2

predict(res2,transf=transf.ztor)

##Sensitivity analyses

l3<-leave1out(res2)

summary(l3$estimate)

## select only positive results to compare summary effect of published significant samples with the p-uniform effect estimate

res3<-rma(yi,vi,data=data,subset=r>-.001)

res3

predict(res3,transf=transf.ztor)

res3.fe<-rma(yi,vi,data=data,subset=r>-.001,method="FE")

res3.fe

predict(res3.fe,transf=transf.ztor)

##subgroup analysis für published vs. unpublished samples

comparison<-data.frame(ES=c(coef(res1),coef(res2)),SE=c(res1$se,res2$se),factor=c(1,2),tau2=round(c(res1$tau2,res2$tau2),3))

rma(ES, sei=SE,mods=~factor,method="FE",data=comparison,digits=3)

## all publication bias methods are applied on published samples only

##trim and fill für published results

trimfill(res1,side="left")

##contour-enhanced funnel plot

funnel(res1,yaxis="seinv",level=c(90, 95, 99), shade=c("white", "gray", "darkgray"), refline=0,pch.fill=21)

abline(v=res1$b,lty=2)

##Egger regression

regtest(res1)

##Begg und Mazumdar

ranktest(res1)

## Separate correlations for initials are available upon request from the first author at jakob.pietschnig@univie.ac.at

#############################

## Subset effect Initial 1 ##

#############################

data.i1<-escalc(ri=r1initial,ni=sample_size,data=c,measure="ZCOR")

res.i1<-rma(yi,vi,data=data.i1)

res.i1.predict<-predict(res.i1,transf=transf.ztor)

#############################

## Subset effect Initial 2 ##

#############################

data.i2<-escalc(ri=r2initial,ni=sample_size,data=c,measure="ZCOR")

res.i2<-rma(yi,vi,data=data.i2)

res.i2.predict<-predict(res.i2,transf=transf.ztor)

#######################

## Subgroup analyses ##

#######################

##zs need to be transformed to rs for interpretation using the predict function (exp(2*z)-1)/(exp(2*z)+1)

##Subgroup analysis pen and paper vs. computer administration

data$administration

res1.adm<-rma(yi,vi,data=data,subset=administration=="pen and paper")

summary(res1.adm)

res2.adm<-rma(yi,vi,data=data,subset=administration=="computer")

summary(res2.adm)

comparison.adm<-data.frame(ES=c(coef(res1.adm),coef(res2.adm)),SE=c(res1.adm$se,res2.adm$se),factor=c(1,2),tau2=round(c(res1.adm$tau2,res2.adm$tau2),3))

rma(ES, sei=SE,mods=~factor,method="FE",data=comparison.adm,digits=3)

##Subgroup analysis algorithm b vs. other algorithms

data$alg_comp

res1.alg<-rma(yi,vi,data=data,subset=alg_comp=="b-algorithm")

summary(res1.alg)

predict(res1.alg,transf=transf.ztor)

res2.alg<-rma(yi,vi,data=data,subset=alg_comp=="other algorithm")

summary(res2.alg)

predict(res2.alg,transf=transf.ztor)

comparison.alg<-data.frame(ES=c(coef(res1.alg),coef(res2.alg)),SE=c(res1.alg$se,res2.alg$se),factor=c(1,2),tau2=round(c(res1.alg$tau2,res2.alg$tau2),3))

rma(ES, sei=SE,mods=~factor,method="FE",data=comparison.alg,digits=3)

##Subgroup analysis unpublished vs. published samples

res1.pub<-rma(yi,vi,data=data,subset=publication_status=="published")

summary(res1.pub)

res2.unpub<-rma(yi,vi,data=data,subset=publication_status=="unpublished")

summary(res2.unpub)

comparison.pub<-data.frame(ES=c(coef(res1.pub),coef(res2.unpub)),SE=c(res1.pub$se,res2.unpub$se),factor=c(1,2),tau2=round(c(res1.pub$tau2,res2.unpub$tau2),3))

rma(ES, sei=SE,mods=~factor,method="FE",data=comparison.pub,digits=3)

##Subgroup analysis BNT (birthday number preference) included vs. not included

res1.bnt<-rma(yi,vi,data=data,subset=bnt=="no")

summary(res1.bnt)

res2.bnt<-rma(yi,vi,data=data,subset=bnt=="yes")

summary(res2.bnt)

comparison.bnt<-data.frame(ES=c(coef(res1.bnt),coef(res2.bnt)),SE=c(res1.bnt$se,res2.bnt$se),factor=c(1,2),tau2=round(c(res1.bnt$tau2,res2.bnt$tau2),3))

rma(ES, sei=SE,mods=~factor,method="FE",data=comparison.bnt,digits=3)

##Subgroup analysis liking vs. beauty

res1.l<-rma(yi,vi,data=data,subset=likingvsbeauty=="liking")

summary(res1.l)

predict(res1.l,transf=transf.ztor)

res2.b<-rma(yi,vi,data=data,subset=likingvsbeauty=="beauty")

summary(res2.b)

predict(res2.b,transf=transf.ztor)

comparison.lvb<-data.frame(ES=c(coef(res1.l),coef(res2.b)),SE=c(res1.l$se,res2.b$se),factor=c(1,2),tau2=round(c(res1.l$tau2,res2.b$tau2),3))

rma(ES, sei=SE,mods=~factor,method="FE",data=comparison.lvb,digits=3)

##Subgroup analysis single vs. double administration

res1.s<-rma(yi,vi,data=data,subset=singlevsdouble=="single")

summary(res1.s)

predict(res1.s,transf=transf.ztor)

res2.d<-rma(yi,vi,data=data,subset=singlevsdouble=="double")

summary(res2.d)

predict(res2.d,transf=transf.ztor)

comparison.svd<-data.frame(ES=c(coef(res1.s),coef(res2.d)),SE=c(res1.s$se,res2.d$se),factor=c(1,2),tau2=round(c(res1.s$tau2,res2.d$tau2),3))

rma(ES, sei=SE,mods=~factor,method="FE",data=comparison.svd,digits=3)

##Subgroup analysis for sequence of administration: expl-impl (order 1) vs. impl-expl (order 2)

res1_seq<-rma(yi,vi,data=data,subset=order==1,slab=authors)

res1_seq

res2_seq<-rma(yi,vi,data=data,subset=order==2,slab=authors)

res2_seq

comparison_seq<-data.frame(ES=c(coef(res1_seq),coef(res2_seq)),SE=c(res1_seq$se,res2_seq$se),factor=c(1,2),tau2=round(c(res1_seq$tau2,res2_seq$tau2),3))

rma(ES, sei=SE,mods=~factor,method="FE",data=comparison_seq,digits=3)

##################################

## Hierarchical Meta-Regression ##

##################################

## First step

## RE model

regr1<-rma(yi,vi,mods=~publication_year,data=data)

regr1

## calculate eta-squared from fixed effect model

lm1<-lm(yi~publication_year,weights=1/sqrt(vi),data=data)

etasq(lm1)

## calculate beta values for regression

regr1_scaled<-rma(yi,vi,mods=~scale(publication_year),data=data)

regr1_scaled

##calculate regression for subset to compare with step 2 (ML)

regr1_ML<-(rma(yi,vi,mods=~publication_year,subset=(!(is.na(male_ratio))),data=data,method="ML"))

summary(regr1_maleratio)

## Second step

## RE model

regr2<-rma(yi,vi,mods=~publication_year+male_ratio,data=data)

regr2

## calculate eta-squared and VIFs from fixed effect model

lm2<-lm(yi~publication_year+male_ratio,weights=1/sqrt(vi),data=data)

summary(lm2)

vif(lm2)

etasq(lm2)

## calculate beta values for regression

regr2_scaled<-rma(yi,vi,mods=~scale(publication_year)+scale(male_ratio),data=data)

regr2_scaled

##calculate ML regression for model comparison (ML)

regr2_ML<-rma(yi,vi,mods=~publication_year+male_ratio,data=data,method="ML")

##compare model 1 with model 2

anova(regr1_ML,regr2_ML)

## Final step

## RE model

regr3<-rma(yi,vi,mods=~publication_year+male_ratio+publication_status,data=data)

regr3

## calculate eta-squared and VIFs from fixed effect model

lm3<-lm(yi~publication_year+male_ratio+publication_status,weights=1/sqrt(vi),data=data)

summary(lm3)

vif(lm3)

etasq(lm3)

## calculate beta values for regression

regr3_scaled<-rma(yi,vi,mods=~scale(publication_year)+scale(male_ratio)+scale(as.numeric(publication_status)),data=data)

regr3_scaled

##calculate ML regression for model comparison (ML)

regr3_ML<-rma(yi,vi,mods=~publication_year+male_ratio+publication_status,data=data,method="ML")

##compare model 2 with model 3

anova(regr2_ML,regr3_ML)

############################

#### Excess significance ###

## Ioannidis & Trikalinos ##

############################

##remove sign. neg. value from data

data_it<-subset(data,(publication_status=="published"))

##calculate overall effect

res_it<-rma(yi,vi,data=data_it)

res_it.predict<-predict(res_it,transf=transf.ztor)

res_it.predict$pred

#Calculating average power based on effect size estimate of overall effect (published effects only)

P.ind.all.e<-mapply(pwr.r.test,n=data_it$sample_size,r=res_it.predict$pred)

PowP.ind.all.e<-sapply(P.ind.all.e[4,1:78], as.numeric)

MeanPowerInd.all.e<-mean(PowP.ind.all.e)

################################################

## average power based on the observed effect ##

################################################

MeanPowerInd.all.e

## the average power * number of studies (78) = expected number of sign. results

e.all.e<-MeanPowerInd.all.e*78

e.all.e

## calculate chi-squared test for observed sign. effects in summary effect direction (24) vs. expected sign. effects (e.all.e)

A<-((24-e.all.e)^2/e.all.e)+((24-e.all.e)^2/(78-e.all.e))

pchisq(A, df=1, lower.tail=F)
