# Supplementary material for: Indirect (implicit) and direct (explicit) self-esteem measures are virtually unrelated: A meta-analysis of the initial preference task
Source: PLoS One. 2018 Sep 6;13(9):e0202873. doi: 10.1371/journal.pone.0202873 (PMC6126831; doi:10.1371/journal.pone.0202873)
Supplement: S1 Table — (DOCX) [file pone.0202873.s003.docx]

Table S3. Characteristics of included samples.

| Authors | Country | Publication status | Mean age | % men | Format | Explicit measure | order | Rating type | algorithm | *r* | *n* |
| --- | --- | --- | --- | --- | --- | --- | --- | --- | --- | --- | --- |
| Bosson et al. (2000) | USA | published |  | 48.19 | computer | RSES | counterbalanced | liking | b-algorithm | 0.13 | 83 |
| Jones et al. (2002)^c^ | USA | published |  | 41.96 | pen and paper | RSES | expl-impl | liking | b-algorithm | ‑0.12 | 143 |
| Bosson et al. (2003) 1 | USA | published |  | 34.48 | pen and paper | SLCS | counterbalanced | liking | b-algorithm | -0.19 | 116 |
| Bosson et al. (2003) 2 | USA | published |  | 29.75 | pen and paper | SLCS-R | impl-expl | liking | b-algorithm | 0.15 | 158 |
| Hodson & Olson (2005)^e^ 1 | Canada | Personal communication |  | 18.75 | pen and paper | RSES | impl-expl | liking | other | 0.03 | 176 |
| Hodson & Olson (2005)^e^ 2 | Canada | Personal communication |  | 17.65 | pen and paper | RSES | impl-expl | liking | other | 0.13 | 187 |
| Lakey (2005) | USA | unpublished |  | 11.88 | pen and paper | RSES | expl-impl | beauty | b-algorithm | 0.26 | 101 |
| Pelham et al. (2005) 1 | Netherlands | published |  | 0.00 | pen and paper | RSES | unclear | liking | b-algorithm | 0.36 | 65 |
| Pelham et al. (2005) 2 | Netherlands | published |  | 100.00 | pen and paper | RSES | unclear | liking | b-algorithm | -0.13 | 34 |
| Pelham et al. (2005) 3^c^ | Singapore | published |  | 0.00 | pen and paper | RSES | unclear | liking | b-algorithm | 0.25 | 85 |
| Pelham et al. (2005) 4^c^ | Singapore | published |  | 100.00 | pen and paper | RSES | unclear | liking | b-algorithm | -0.15 | 34 |
| Pelham et al. (2005) 5 | USA | published |  | 0.00 | pen and paper | RSES | unclear | liking | b-algorithm | 0.11 | 412 |
| Pelham et al. (2005) 6 | USA | published |  | 100.00 | pen and paper | RSES | unclear | liking | b-algorithm | 0.09 | 230 |
| DeHart et al. (2006) 1^d^ | USA | published | 21.25 | 30.82 | pen and paper | RSES | expl-impl | liking | b-algorithm | 0.15 | 159 |
| DeHart et al. (2006) 2^c^ | USA | published | 19.50 | 37.65 | mixed | RSES | expl-impl | liking | b-algorithm | 0.22 | 85 |
| DeHart et al. (2006) 3^d^ | USA | published | 21.90 | 38.51 | pen and paper | RSES | expl-impl | liking | b-algorithm | 0.18 | 309 |
| Zeigler-Hill (2006) | USA | published |  | 30.83 | pen and paper | RSES | impl-expl | liking | b-algorithm | 0.09 | 120 |
| Karpinski et al. (2007) 1 | USA | published |  | 26.92 | computer | RSES (composite) | impl-expl | liking | z-algorithm | 0.15 | 52 |
| Karpinski et al. (2007) 2 | USA | published |  | 25.42 | computer | RSES | impl-expl | liking | z-algorithm | 0.37 | 86 |
| Lima (2007) | USA | unpublished | 19.42 | 55.21 | computer | RSES | unclear | liking | b-algorithm | -0.05 | 259 |
| Schroeder-Abe et al. (2007) | Germany | published | 22.69 | 21.57 | computer | MSES | impl-expl | liking | s-algorithm | -0.12 | 102 |
| Steinberg et al. (2007) 1 | USA | published |  |  | unclear | RSES | impl-expl | beauty | z-scores | 0.28 | 98 |
| Steinberg et al. (2007) 2 | USA | published |  |  | unclear | RSES | impl-expl | beauty | z-scores | 0.03 | 83 |
| Zeigler-Hill & Terry (2007) | USA | published | 19.37 | 40.14 | pen and paper | RSES | expl-impl | liking | b-algorithm | 0.10 | 563 |
| Good (2008) | USA | unpublished | 18.82 | 42.11 | computer | RSES | unclear | liking | b-algorithm | 0.03 | 361 |
| Kernis et al. (2008) | USA | published |  | 11.88 | unclear | RSES | expl-impl | beauty | b-algorithm | 0.24 | 101 |
| Krizan (2008) 1 | USA | published |  |  | pen and paper | SLSC | impl-expl | likiing | b-algorithm | 0.25 | 42 |
| Krizan (2008) 2 | USA | published |  |  | pen and paper | SLSC | impl-expl | liking | b-algorithm | -0.05 | 59 |
| Rudolph et al. (2008)^b^ | Germany | published | 22.40 | 66.10 | computer | MSES | impl-expl | liking | b-algorithm | 0.11 | 59 |
| Albers et al. (2009) 1 | Netherlands | published |  | 29.95 | computer | SSES | counterbalanced | beauty | r-algorithm | 0.15 | 384 |
| Albers et al. (2009) 2 | Netherlands | published |  | 35.94 | computer | RSES | impl-expl | beauty | r-algorithm | 0.17 | 128 |
| Albers et al. (2009) 3 | Netherlands | published |  | 31.03 | computer | RSES | impl-expl | beauty | r-algorithm | 0.16 | 58 |
| Dandeneau & Baldwin (2009) | Canada | published | 22.00 | 44.67 | computer | RSES | expl-impl | liking | unclear | -0.01 | 150 |
| DeHart et al. (2009) | USA | published | 18.80 | 47.52 | computer | RSES | unclear | Liking | b-algorithm | 0.21 | 505 |
| Koole et al. (2009) 1 | USA | published | 19.00 | 41.54 | computer | RSES | impl-expl | liking | b-algorithm | -0.07 | 65 |
| Koole et al. (2009) 2 | USA | published | 19.00 | 41.54 | computer | RSES | impl-expl | liking | b-algorithm | 0.27 | 65 |
| Koole et al. (2009) 3 | Netherlands | published | 21.00 | 17.65 | computer | RSES | impl-expl | beauty | b-algorithm | -0.07 | 34 |
| Koole et al. (2009) 4 | Netherlands | published | 21.00 | 16.67 | computer | RSES | impl-expl | beauty | b-algorithm | 0.49 | 24 |
| LeBel & Gawronski (2009) 1 | Canada | published | 18.50 | 31.87 | computer | RSES | unclear | unclear | b-algorithm | 0.30 | 91 |
| LeBel & Gawronski (2009) 2 | Canada | published | 19.40 | 18.52 | computer | RSES | unclear | unclear | b-algorithm | 0.24 | 81 |
| LeBel & Gawronski (2009) 3 | Canada | published | 18.90 | 60.53 | computer | RSES | expl-impl | liking | b-algorithm | 0.03 | 76 |
| LeBel & Gawronski (2009) 4 | Canada | published | 22.90 | 24.05 | computer | RSES | impl-expl | beauty | b-algorithm | 0.08 | 79 |
| LeBel & Gawronski (2009) 5 | Canada | published | 18.80 | 27.27 | computer | RSES | expl-impl | liking | b-algorithm | 0.13 | 77 |
| LeBel & Gawronski (2009) 6 | Canada | published | 19.00 | 45.00 | computer | RSES | expl-impl | liking | b-algorithm | 0.06 | 80 |
| LeBel & Gawronski (2009) 7 | Canada | published | 18.50 | 30.77 | computer | RSES | expl-impl | liking | b-algorithm | 0.16 | 78 |
| LeBel & Gawronski (2009) 8 | Canada | published | 18.90 | 48.75 | computer | RSES | impl-expl | liking | b-algorithm | 0.05 | 80 |
| LeBel & Gawronski (2009) 9 | Canada | published | 21.80 | 26.27 | computer | RSES | impl-expl | liking | b-algorithm | 0.07 | 118 |
| LeBel & Gawronski (2009) 10 | Canada | published | 18.80 | 22.86 | computer | RSES | impl-expl | liking | b-algorithm | -0.03 | 70 |
| LeBel & Gawronski (2009) 11 | Canada | published | 18.80 | 30.07 | computer | RSES | counterbalanced | liking | b-algorithm | 0.18 | 725 |
| LeBel & Gawronski (2009) 12 | Canada | published | 28.10 | 31.79 | computer | RSES | counterbalanced | liking | b-algorithm | 0.21 | 324 |
| LeBel & Gawronski (2009) 13 | Canada | published | 18.60 | 31.76 | computer | RSES | unclear | unclear | b-algorithm | 0.09 | 85 |
| LeBel & Gawronski (2009) 14 | Canada | published | 31.30 | 40.38 | computer | RSES | unclear | unclear | b-algorithm | 0.22 | 260 |
| LeBel & Gawronski (2009) 15 | Canada | published | 31.50 | 34.93 | computer | RSES | counterbalanced | liking | b-algorithm | 0.29 | 272 |
| Tracy et al. (2009) 1 | Canada | published | 19.20 | 38.78 | computer | RSES | Counterbalanced | liking | i-algorithm | 0.05 | 49 |
| Tracy et al. (2009) 2 | Canada | published | 19.40 | 44.19 | computer | RSES | counterbalanced | beauty | i-algorithm | 0.12 | 43 |
| Gregg & Sedikides (2010) | UK | published | 20.50 | 15.00 | computer | RSES | expl-impl | liking | i-algorithm | -0.03 | 115 |
| LeBel (2010) | Canada | published | 20.90 | 23.00 | computer | RSES | counterbalanced | liking | b-algorithm | 0.14 | 200 |
| Peterson (2010) | USA | unpublished | 20.73 | 50.00 | pen and paper | RSES | unclear | liking | b-algorithm | 0.14 | 204 |
| Stieger & Burger (2010)^e^ 1 | Austria | published | 27.10 | 43.00 | pen and paper | RSES | random | liking | i-algorithm | -0.02 | 244 |
| Stieger & Burger (2010)^e^ 2 | Austria | published | 26.10 | 42.00 | pen and paper | RSES | impl-expl^a^ | liking | i-algorithm | 0.09 | 138 |
| Eichinger (2011)^e^ | Austria | unpublished | 33.72 | 58.00 | pen and paper | RSES | impl-expl^a^ | liking | i-algorithm | -0.04 | 100 |
| Huntsinger (2011) | USA | published |  | 20.71 | pen and paper | RSES | impl-expl | liking | i-algorithm | 0.06 | 140 |
| Krause et al. (2011) | Germany | published | 23.50 | 22.77 | computer | RSES | expl-impl | liking | i-algorithm | 0.21 | 101 |
| Sava et al. (2011) | Romania | published | 20.50 | 14.71 | pen and paper | RSES | counterbalanced | liking | b-algorithm | 0.17 | 102 |
| Shimizu & Pelham (2011)^c^ | USA | published |  | 50.00 | pen and paper | RSES | unclear | liking | b-algorithm | 0.10 | 110 |
| Trask-Tolbert (2011) | USA | unpublished |  |  | pen and paper | RSES | unclear | liking | i-algorithm | 0.00 | 123 |
| Zeigler-Hill et al. (2011) | USA | published | 19.58 | 29.51 | pen and paper | RSES | expl-impl | liking | b-algorithm | 0.07 | 288 |
| Creemers et al. (2012) | Netherlands | published | 21.20 | 0.00 | computer | RSES | impl-expl | liking | s-algorithm | -0.08 | 95 |
| Falk (2012) 1 | Canada | unpublished | 20.37 | 25.56 | computer | RSES | impl-expl | liking | i-algorithm | -0.07 | 180 |
| Falk (2012) 2 | North America | unpublished | 32.25 | 34.88 | computer | RSES | impl-expl | liking | i-algorithm | 0.09 | 582 |
| Gruseck (2012)^e^ 1 | Austria | unpublished | 24.26 | 47.22 | pen and paper | RSES | counterbalanced | liking | i-algorithm | -0.10 | 216 |
| Gruseck (2012)^e^ 2 | Spain | unpublished | 23.00 | 44.27 | pen and paper | RSES | counterbalanced | liking | i-algorithm | 0.06 | 253 |
| Phillips et al. (2012) | Australia | published | 29.91 | 18.63 | computer | RSES | impl-expl | beauty | z-algorithm | 0.12 | 306 |
| Ryan (2012) | USA | unpublished |  | 34.05 | computer | RSES | random | beauty | i-algorithm | 0.03 | 420 |
| Stieger et al. (2012)^e^ 1 | Austria | published | 34.30 | 100.00 | pen and paper | RSES | impl-expl^a^ | liking | i-algorithm | 0.11 | 89 |
| Stieger et al. (2012)^e^ 2 | Austria | published | 34.30 | 0.00 | pen and paper | RSES | impl-expl^a^ | liking | i-algorithm | 0.04 | 65 |
| Zeigler-Hill et al. (2012) | USA | published | 20.49 | 23.33 | pen and paper | RSES | expl-impl | liking | b-algorithm | 0.11 | 210 |
| Huntsinger (2013a) | USA | published |  | 19.00 | pen and paper | RSES | impl-expl | liking | i-algorithm | 0.13 | 100 |
| Huntsinger (2013b) | USA | published |  | 25.56 | pen and paper | RSES | impl-expl | liking | i-algorithm | 0.23 | 90 |
| Peterson & DeHart (2013) 1 | USA | Personal communication | 20.40 | 35.16 | computer | RSES | expl-impl | liking | b-algorithm | 0.27 | 128 |
| Peterson & DeHart (2013) 2 | USA | Personal communication | 20.73 | 50.00 | computer | RSES | expl-impl | liking | b-algorithm | 0.28 | 204 |
| Randolph-Seng & Gardner (2013) | USA | published | 18.91 | 37.40 | computer | RSES | random | beauty | b-algorithm | 0.05 | 131 |
| Stieger & Burger (2013)^e^ 1 | Austria | published | 29.10 | 42.67 | pen and paper | RSES | impl-expl^a^ | liking | i-algorithm | 0.13 | 75 |
| Stieger & Burger (2013)^e^ 2 | Austria | published | 29.10 | 42.06 | pen and paper | RSES | impl-expl^a^ | liking | i-algorithm | -0.13 | 126 |
| Bodroza (2014)^e^ | Serbia | published | 20.10 | 17.92 | pen and paper | GSES | unclear | liking | i-algorithm | 0.07 | 279 |
| Bujak (2014) | USA | unpublished | 20.11 | 0.00 | pen and paper | RSES | counterbalanced | liking | b-algorithm | 0.03 | 61 |
| Hoorens (2014) | Netherlands | published | 18.40 | 17.54 | pen and paper | SLSC | unclear | beauty | i-algorithm | 0.07 | 342 |
| Peterson (2014) 1 | USA | Personal communication |  |  | computer | SSES | impl-expl | liking | b-algorithm | 0.42 | 35 |
| Peterson (2014) 2 | USA | Personal communication |  |  | computer | SSES | impl-expl | liking | b-algorithm | 0.09 | 31 |
| Peterson (2014) 3 | USA | Personal communication |  |  | computer | SSES | impl-expl | liking | b-algorithm | 0.16 | 48 |
| Peterson (2014) 4 | USA | Personal communication |  |  | computer | SSES | impl-expl | liking | b-algorithm | -0.23 | 46 |
| Peterson (2014) 5 | USA | Personal communication |  |  | computer | SSES | impl-expl | liking | b-algorithm | 0.01 | 47 |
| Saryiska et al. (2014) 1 ^e,f^ | Germany | Personal communication |  |  | mixed | RSES | unclear | liking | other | 0.25 | 65 |
| Saryiska et al. (2014) 2 ^e,f^ | Bulgaria | Personal communication |  |  | computer | RSES | unclear | liking | other | 0.07 | 138 |
| Saryiska et al. (2014) 3 ^e,f^ | Spain | Personal communication |  |  | computer | RSES | unclear | liking | other | -0.01 | 118 |
| Saryiska et al. (2014) 4 ^e,f^ | Colombia | Personal communication |  |  | computer | RSES | unclear | liking | other | 0.04 | 74 |
| Stieger et al. (2014)^e^ | Austria | published | 37.70 | 42.00 | pen and paper | RSES | impl-expl | liking | i-algorithm | -0.01 | 1765 |
| Hoorens et al. (2015)^e^ | Belgium | published | 34.57 | 35.37 | pen and paper | RSES | expl-impl | beauty | b-algorithm | 0.30 | 164 |
| Kim & Johnson (2015) | USA | Personal communication | 19.57 | 37.50 | pen and paper | RSES | expl-impl | beauty | b-algorithm | 0.11 | 40 |
| Otto (2015) | USA | unpublished | 32.80 | 49.31 | computer | RSES | unclear | liking | b-algorithm | 0.28 | 290 |
| Rusu et al. (2015) | Romania | published | 20.70 | 50.00 | computer | RSES | unclear | liking | i-algorithm | 0.01 | 212 |
| Krause et al. (2016) | Germany | published | 24.10 | 44.89 | computer | RSES | expl-impl | liking | i-algorithm | 0.04 | 127 |
| Maroui et al. (2016) | Romania | published | 23.10 | 44.55 | pen and paper | RSES | expl-impl | liking | i-algorithm | 0.15 | 110 |
| Smallets et al. (2016) | USA | published |  | 34.86 | computer | RSES | unclear | liking | b-algorithm | 0.12 | 109 |
| Hamilton & DeHart (2017) | USA | published | 20.42 | 33.00 | computer | RSES | unclear | liking | b-algorithm | 0.08 | 178 |

*Note*. When multiple studies were presented within the same paper, we numbered included samples according to the sequence of included studies; Personal communications refer to published papers where correlation coefficients were not explicitly reported but obtained through correspondence; ^a^indicates double administration of the IPT once preceding and once subsequent to the explicit measure: within analyses these cases were treated as implicit-explicit administrations; ^b^indicates double administration of the IPT before or after the explicit measure was administered; ^c^indicates that the IPT was a compound measure including birthday number preference; ^d^indicates that both measures have been administered at multiple time-points and respective results were therefore averaged; ^e^indicates that first and last name IPT scores were provided separately; ^f^indicates triple administration of the IPT; blank cells indicate that no information was retrievable from primary studies; *r* = associations between Initial Preference Task and explicit self-esteem measures; GSES = General Self Esteem Scale; MSES = Multidimensional Self Esteem Scale; RSES = Rosenberg Self Esteem Scale; SLCS/SLSC = Self-Liking Self-Competence Scale; SSES = State Self Esteem Scale; impl-expl = Implicit measure was administered first; expl-impl = explicit measure was administered first.
